# Genome sequencing of biocontrol strain *Bacillus amyloliquefaciens* Bam1 and further analysis of its heavy metal resistance mechanism

**DOI:** 10.1186/s40643-022-00563-x

**Published:** 2022-07-18

**Authors:** Yuanchan Luo, Lei Chen, Zhibo Lu, Weijian Zhang, Wentong Liu, Yuwei Chen, Xinran Wang, Wei Du, Jinyan Luo, Hui Wu

**Affiliations:** 1 Department of Applied Biology, School of Biotechnology, East China University of Science and Technology, 130 Meilong Road, Shanghai, 200237 China; 2 Department of Plant Quarantine, Shanghai Extension and Service Center of Agriculture Technology, Shanghai, 201103 China; 3 Agricultural Technology Extension Station of Ningxia, 2, West Shanghai Road, Yinchuan, 750001 China; 4 State Key Laboratory of Bioreactor Engineering, School of Biotechnology, East China University of Science and Technology, 130 Meilong Road, Shanghai, 200237 China; 5 Shanghai Collaborative Innovation Center for Biomanufacturing Technology, 130 Meilong Road, Shanghai, 200237 China; 6 Key Laboratory of Bio-Based Material Engineering of China National Light Industry Council, 130 Meilong Road, Shanghai, 200237 China

**Keywords:** *Bacillus amyloliquefaciens*, Heavy metal resistance mechanism, Comparative genomic analysis, Resistance to essential heavy metals, Resistance to non-essential heavy metals

## Abstract

Plant growth-promoting rhizobacteria (PGPR) or Biocontrol strains inevitably encounter heavy metal excess stress during the product’s processing and application. *Bacillus amyloliquefaciens* Bam1 was a potential biocontrol strain with strong heavy metal resistant ability. To understand its heavy metal resistance mechanism, the complete genome of Bam1 had been sequenced, and the comparative genomic analysis of Bam1 and FZB42, an industrialized PGPR and biocontrol strain with relatively lower heavy metal tolerance, was conducted. The comparative genomic analysis of Bam1 and the other nine *B. amyloliquefaciens* strains as well as one *Bacillus velezensis* (genetically and physiologically very close to *B. amyloliquefaciens*) was also performed. Our results showed that the complete genome size of Bam1 was 3.95 Mb, 4219 coding sequences were predicted, and it possessed the highest number of unique genes among the eleven analyzed strains. Nine genes related to heavy metal resistance were detected within the twelve DNA islands of Bam1, while only two of them were detected within the seventeen DNA islands of FZB42. When compared with *B. amyloliquefaciens* type strain DSM7, Bam1 lacked contig L, whereas FZB42 lacked contig D and I, as well as just possessed contig B with a very small size. Our results could also deduce that Bam1 promoted its essential heavy metal resistance mainly by decreasing the import and increasing the export of heavy metals with the corresponding homeostasis systems, which are regulated by different metalloregulators. While Bam1 promoted its non-essential heavy metal resistance mainly by the activation of some specific or non-specific exporters responding to different heavy metals. The variation of the genes related to heavy metal resistance and the other differences of the genomes, including the different number and arrangement of contigs, as well as the number of the heavy metal resistant genes in Prophages and Genomic islands, led to the significant different resistance of Bam1 and FZB42 to heavy metals.

**Graphical Abstract:**

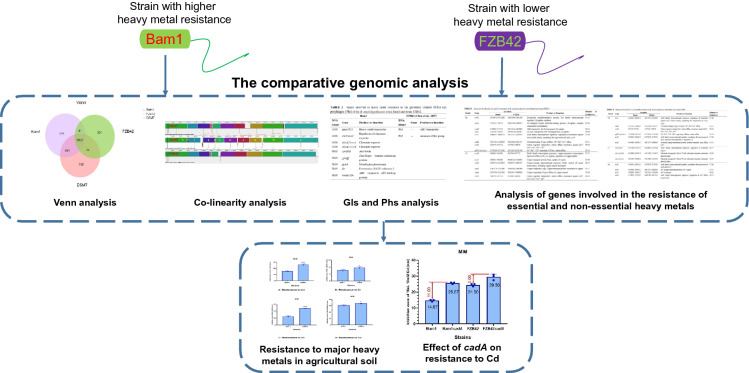

**Supplementary Information:**

The online version contains supplementary material available at 10.1186/s40643-022-00563-x.

## Introduction

*Bacillus amyloliquefaciens* is a typical Gram-positive plant growth-promoting rhizobacteria (PGPR) (Chen et al. 2007; Fan et al. 2018; Ravi et al. 2021). Simultaneously, it is also an excellent and important biocontrol agent (Fan et al. 2018; Gautam et al. 2019). Many strains of *B. amyloliquefaciens* have been registered and commercialized as biopesticides to control plant diseases all over the world (CPIN;^1^ EPA^2^; Fan et al. 2018). *B. velezensis* is initially identified as *B. amyloliquefaciens* and recently classified in the *B. velezensis* species as well as still remains genetically and physiologically very close to its ancestor *B. amyloliquefaciens* (Chun et al. 2019)*.* Therefore, many *B. velezensis* strains are also potential biocontrol strains and RGPRs (Silva et al. 2019). Among the RGPRs and the biocontrol strains from *B. amyloliquefaciens* and *B. velezensis*, *B. velezensis* FZB42 strain (previously identified as *B. amyloliquefaciens*) is the most famous one for the commercialization of the products derived from it and its ‘cousin’ strain (Fan et al. 2018). The intensive research on the mechanisms of plant-growth-promoting and plant disease biocontrolling of the FZB42 strain was also wildly reported. In addition, a ‘Subwiki’-like data bank, ‘AmyloWiki’, has been built up, which contains accumulated information about the genes that are presented in strain FZB42 (Fan et al. 2018).

‘Heavy metal’ means the elements with density greater than 6 g/cm^3^ (Hooda 2010). Some heavy metals are essential micronutrients of microorganisms. They are usually cofactors of many important enzymes and structural components of some proteins, involved in different metabolism for maintaining the life process in microorganisms (Moore and Helmann 2005). These essential trace elements include manganese (Mn), iron (Fe), zinc (Zn), copper (Cu), and cobalt (Co) (Hooda 2010; Mu and Zhu 2019). Though the essential trace metal ions are crucial for the growth or other physiological functions of microorganisms, however, metal excess can be toxic (Moore et al. 2005; Chandrangsu et al. 2017; Huang et al. 2017a; Sachla et al. 2021). Metal ion intoxication may result from the mismetallation of essential metal-dependent enzymes with a noncognate metal. Enzyme mismetallation can impair metabolism, lead to new and deleterious reactions, and cause cell death (Pi et al. 2020). Enzymes may bind to the wrong metal cofactor when one metal is at toxic levels. For example, Mn^2+^ or Co^2+^ intoxication results from disruption of an Mg^2+^-dependent enzyme or process (Pi et al. 2020). When Zn^2+^ is mismetallated with PerR, a protein originally metallated with Fe^2+^ or Mn^2+^ will result in oxidative stress in the cell (Chandrangsu and Helmann 2016). Excess Fe^2+^ also leads to oxidative stress in the cell (VanderWal et al. 2017). While excess Cu^2+^ is hazardous to cellular processes and macromolecules, due to the effects of localized free-radical damage (Sullivan et al. 2021). When the PGPRs or biocontrol strains are developed together with micronutrients into microbial fertilizer, they will inevitably face metal excess, because the critical trace metals (Mn, Fe, Zn, Cu, Co) for microbes are also essential for the growth or other functions of most plants (Alloway 2008). For application with an appropriate concentration infield, the concentration of these essential heavy metals in the preparation should be concentrated, which leads to a metal excess condition for the PGPRs or biocontrol strains. For survival in adverse metal conditions, metal starvation, or excess, the microorganisms can form a delicate balance of the uptake and efflux systems to keep the metal ions homeostasis in their cells (Moore and Helmann 2005). Some of these systems, for example, Mn uptake and efflux pumps regulated by a bifunctional regulator, MntR, have been revealed in *Bacillus subtilis* (Huang et al. 2017a). For some other heavy metals, such as cadmium (Cd), chromium (Cr), lead (Pb), mercury (Hg), and arsenic (As, metalloid), there is little evidence to suggest that they play a nutritive role in microorganisms (Hooda 2010). While with the rapid development of the mining and manufacturing industry, as well as the misuse of chemical fertilizers and pesticides, many agricultural soils have been contaminated by heavy metals (Hooda 2010; Huang et al. 2017b; Bulletin of investigation on soil pollution in China^3^). The contaminated heavy metals that have been detected in the cultivated lands include the non-essential elements, Cd, Cr, Pb, Hg, and As, as well as the essential elements, Cu, and Zn (Hooda 2010; Huang et al. 2017b; Islam et al. 2018). Cd is the primary pollutant in the agricultural soil in many countries (e.g., Japan, China, Korea, Iran, and Australia et.) (Yuan et al. 2013; Huang et al. 2017b; Yazdia et al. 2019). However, most contaminated land is still at a light-polluted level and some researchers declared that the content of the heavy metals within the crops on this kind of land does no obvious harm to the human body (Yuan et al. 2013; Wang et al. 2020). Given the shortage of cultivated land in China or some other Asian countries, this kind of lightly metal-polluted land is still an indispensable agricultural production resource in these countries. Therefore, the PGPRs or biocontrol strains still face metal excess pressure when they are applied in this kind of cultivated land to enhance the growth of crops or control plant diseases. However, few of the registered PGPRs or biocontrol strains have been screened for their metal resistance. Therefore, it is urgent to screen heavy metal tolerant PGPRs or biocontrol strains and further understand their heavy metal resistant mechanism, which will be beneficial to the product development of microbial pesticide or microbial fertilizer applied in these vast amounts of lightly metal-polluted cultivated land. However, there is a poorly understood resistance mechanism to non-essential heavy metals of PGPRs or biocontrol strains, especially in *B. amyloliquefaciens* and *B. velezensis.*

Genomic comparison is a very useful method for understanding the mechanism of microbes on biocontrol and growth-promoting (Chen et al. 2007; Luo et al. 2018; Yi et al. 2019), heavy metal and antibiotic resistance (Gendy et al. 2020), as well as metabolism or other functions (Li et al. 2019). The whole genomes, including the complete and draft genomes, of about 102 *B. amyloliquefaciens* strains have been sequenced and published in NCBI. The functions of half of these strains have not been published. Most of the strains with published functions are PGPRs or/and biocontrol strains. The research on the comparative genomic analysis of *B. amyloliquefaciens* focus on classification status (Borriss et al. 2011), the principle of metabolism (Chun et al. 2019), biocontrol mechanism (Kröber et al. 2016), as well as synthesis or activity of active substances (Zhai et al. 2019; Choi et al. 2020). However, none of the research involved in heavy metal resistance, and none of the *B. amyloliquefaciens* strains with published complete genome sequence possessed heavy metal resistance research background.

A potential PGPR and biocontrol strain named Bam1 (Additional file 1: Figs. S1, S2) was isolated by our team from the cucumber rhizosphere of a suburb of Beihai, Guangxi, China, and was identified as *B. amyloliquefaciens* by the China General Microbiological Culture Collection Center (CGMCC) with a strain ID of CGMCC 21633. It was found by chance that Bam1 possessed strong resistance to Cd, the main contaminated heavy metal in Chinese agricultural soil.^4^ Since no pesticides or PGPR products have been developed from strains with high heavy metal resistance or tolerance thus far in China, we are developing microbial pesticide and microbial fertilizer containing Bam1, which could be applied to Cd or other heavy metals lightly contaminated cultivated land. Here, the complete genome of Bam1 was sequenced and compared with those of nine other *B. amyloliquefaciens* PGPR or biocontrol strains and *B. velezensis* strain FZB42. To elucidate the difference in the heavy metal resistant mechanism between the strains with strong or weak heavy metal resistance, the main genes involved in different heavy metal resistant mechanisms in Bam1 were compared with those in biocontrol and PGPR strain FZB42. The resistances of strain Bam1and FZB42 to the main heavy metal pollutants (Cd, Cr, Zn and Cu) in Chinese agricultural soil were evaluated. The resistances of these two strains and their key gene deletion mutants to Cd were also tested. This study provided a scientific basis for the further development and application of the bio-products derived from Bam1.

## Materials and methods

### Strains and mutants

*Bacillus amyloliquefaciens* strain Bam1 was isolated from the rhizosphere of cucumber plants in the suburbs of Beihai, Guangxi, China, and deposited in the China General Microbiological Culture Collection Center (CGMCC 21633). The strain was a potential PGPR and biocontrol agent (Additional file 1: Figs. S1, S2). *Bacillus velezensis* strain FZB42 (DSM 23117, named *B. amyloliquefaciens* FZB42 previously) was a commercialized PGPR and biocontrol strain. The *cadA* deletion mutants of Bam1 and FZB42 (Bam1*cadA* and FZB42*cadA*) were constructed by homologous recombination with plasmid pRN5101-Kan. The *qox* deletion mutants of Bam1 and Bam1*cadA* (Bam1*qoxA*, Bam1*qoxB*, Bam1*qoxC*, and Bam1*cadAqoxA*, Bam1*cadAqoxB*, Bam1*cadAqoxC*) were also constructed by the same method.

### Genomic DNA preparation

Strain Bam1 was cultured at 37 °C in Luria–Bertani broth. Genomic DNA was purified from overnight liquid cultures (OD600 nm ≈ 0.8) using the cetyltrimethylammonium bromide method (Watanabe et al. 2010). A TBS-380 fluorometer (Turner BioSystems, United States) or NanoDrop 2500 (Thermo Scientific, United States) was applied to ensure the DNA quality (≥ 10 µg, without degradation, OD260/OD280 ≈ 1.8–2.0).

### Sequencing and assembly

The whole genome was sequenced using the PacBio RS II platform with a 10-kb library. Reads were assembled using SOAPdenovo 2.04^5^ and SPAdes (Bankevich et al. 2012). The assembly data for the complete genome have been deposited in GenBank with the accession number CP082279.

### Genome components and genome annotation

Coding DNA sequence (CDS) prediction was performed using Glimmer 3.02 (Delcher et al. 1999). A circular map of the genome was obtained using Circos version 0.69–6 (Krzywinski et al. 2009). tRNA and rRNA were predicted using the tRNAscan-SEv1.3.1 (Chan and Lowe 2019) and Barrnap 0.8 (Liu et al. 2019), respectively. Genomic islands (GIs) were predicted using IslandViewer 4 (Bertelli et al. 2017). Prophage were found using PHAST (Arndt et al. 2019). Clustered regularly interspaced short palindromic repeat sequences (CRISPRs) were found by CRISPRFinder (Grissa et al. 2007). Functional annotation was based on BLASTN searches (BLAST 2.2.28+) against the NCBI non-redundant (NR) database, Pfam database, EggNOG database, Gene Ontology (GO) database, and Swiss-Prot database.

### Genome comparison

The complete genome sequences of the nine *B. amyloliquefaciens* strains (type strain: DSM7, Biocontrol strains: WF02, ZJU1, SH-B74, T-5, B15, and WS-8, as well as PGPR strains: Y2 and YP6) and one *B. velezensis* strain (FZB42) analyzed in this study were obtained from GenBank. Phylogenetic analysis was conducted for *B. amyloliquefaciens* strains (including *B. velezensis* strain FZB42) inferred by analyzing homologous genes. The single-copy genes of each strain were selected for multiple sequence alignment and quality control comparisons. Multiple sequence alignment was conducted by MAFFT software7.429 (Luo et al. 2018). Quality control comparison was conducted by Gblocks software6 (Luo et al. 2018). The optimal replacement model of the phylogenetic tree was predicted by jModelTest (Posada 2008). Then the phylogenetic tree was constructed by IQ-TREE V1.6.12 based on single-copy gene with 1000 bootstraps (Nguyen et al. 2015). Pan-genome analysis was conducted for the nine other *B. amyloliquefaciens* strains, FZB42, and Bam1. The pan-genome analysis was performed using the OrthoMCL software (Chen et al. 2006). Nucleic acid co-linearity was conducted for the *B. amyloliquefaciens* strains DSM7, FZB42 and Bam1 using the MUMmer 3.0 software (Kurtz et al. 2004).

The core genes involved in the different metal resistant mechanisms were compared between strain Bam1 (with relatively higher heavy metal resistance) and FZB42 (with relatively lower heavy metal resistance). The genes analyzed for the different heavy metal resistance in *B. amyloliquefaciens* were retrieved from the KEGG database, Swiss-Prot database, SubtiWiki database^6^ and Amylowiki database,^7^ as well as selected based on previous studies (Moore and Helmann 2005; Moore et al. 2005; Harvie et al. 2006; Smaldone et al. 2012; Jaroslawiecka and Zofia 2014; Chandrangsu et al. 2017; Huang et al. 2017a; Pi and Helmann 2017; He et al. 2018; Sachla et al. 2021). BLAST was used to compare the identities of the genes between strain Bam1 and FZB42.

### Evaluation of heavy metal resistance

Heavy metal resistances were monitored by disk diffusion (zone-of-inhibition) assay or MIC assay with Minimum media (MM media, Sachla et al. 2021). The disk diffusion assay was carried out according to Sachla et al. (2021). The tested concentration of Cd, Cr, Zn, and Cu were 10 mM, 100 mM, 2 M, and 200 mM, respectively. The resistance of strains Bam1 and FZB42 to Cd, Cr, Zn, and Cu were evaluated. The evaluation of the resistance of strains Bam1, Bam1*cadA*, FZB42, and FZB42*cadA* to Cd was also performed. The evaluation of the Cd resistance of strains Bam1 and its *cadA* and *qox* mutants were also carried out. Three plates for each treatment.

Minimum lethal concentration (MIC) of heavy metals to strains was determined by the Bioscreen method. Tested strains were inoculated in LB broth and grown to an OD 600 nm about 0.4. The culture (5 ml for each culture) was pelleted at 5000 rpm for 5 min at room temperature. The pellet was re-suspended and washed with 5 ml sterile water one time as well as 1× liquid MM two times. The inoculum (re-suspended with 5 mL 1× liquid MM) was added to the wells of a 100 microtiter plate (200 µL per well), 20 wells per treatment, 200 µL serially diluted heavy metal solution was mixed with the inoculum (the last well without heavy metal). The cells were grown under shaking condition for 24–36 h at 28 °C in a Bioscreen C plate reader (Thermo Fisher Scientific, FP-1100-C) and OD 600 nm was monitored. The concentration of Cd in the first well was 20 µM. The tested strain included Bam1, Bam1*cadA*, FZB42, and FZB42*cadA.*

## Results

### Genome features of strain Bam1

**Fig. 1:**
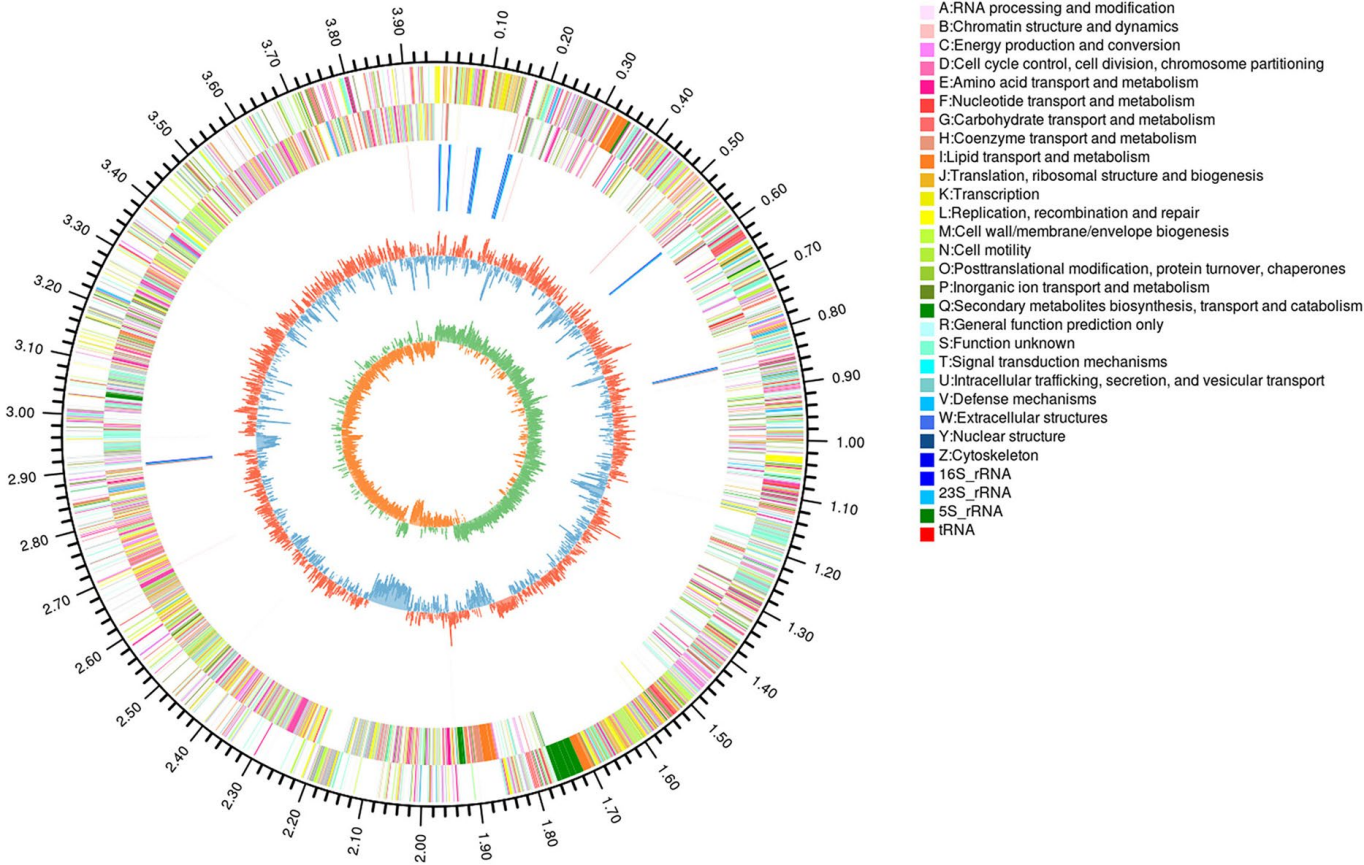
Circular map of the genome of the *B. amyloliquefaciens* strain Bam1. The distribution of the circle from the outside to the inside indicates the genome size, forward CDS, reverse CDS, repeat sequences, tRNA (blue), rRNA (purple), GC ratio (reddish-orange and blue indicate regions, where the GC ratio is higher than average and lower than average, respectively), and GC skew (cyan and orange indicate regions, where the G content is greater than and less than the C content, respectively)

The principal features of the *B. amyloliquefaciens* Bam1 genome are summarized in Table 1. The circular chromosome of strain Bam1 is 3,954,399 bp (Fig. 1), which was somewhat bigger than that of *B. velezensis* FZB42 (3,918,589 bp) (Chen et al. 2007). The average GC content of the chromosome of strain Bam1 was 45.85%. A total of 4219 CDSs, as well as 27 rRNAs and 86 tRNAs, were predicted in the genome of Bam1. While only 3693 CDSs are predicted in strain FZB42 (Chen et al. 2007), which were less than those of strain Bam1.

**Table 1:** Genomic features of the *B. amyloliquefaciens* strain Bam1 and *B. velezensis* strain FZB42

Attribute	Bam1	FZB42 (Chen et al. 2007)
Genome size (bp)	3,954,399	3,918,589
G+C ratio (%)	45.85	46.40
Protein-coding genes	4219	3693
Gene total length (bp)	3,493,872	–
rRNA	27	–
tRNA	86	89
Genomic islands	7	12
Prophage	5	5
CRISPR	7	–

### Genomic islands, prophage, and CRISPR prediction of strain Bam1

The CRISPRs can confer resistance to exogenous genetic elements, such as phages and plasmids (Didovyk et al. 2016; Luo et al. 2018). Seven CRISPRs were detected in Bam1 genome.

Genomic islands (GIs) often carry genes important for genome evolution and adaptation to niches (Lu and Leong 2016). Prophages (Phs) could also enhance the adaptability of bacteria to the environment for the existence of some functional genes in their sequence (Fouts 2006). There seven GIs and five Phs were detected in strain Bam1, while twelve GIs and five Phs were predicted in strain FZB42 (Table 1). Some DNA islands of strain Bam1 showed close relation with the heavy metal resistance. Because nine genes involved in heavy metal resistance were predicted from GI03 and GI06, as well as Ph02, Ph03, and Ph05 in strain Bam1 (Table 2). For example, three genes related to chromate export (encoding one exporting regulator, ChrS, and two exporters, ChrA1 as well as ChrA2) were detected in GI06. The product of *spoIISB* in Ph02 was a stage II sporulation protein SB, which was also an anti-toxin. The product of *pphA* in Ph03 was a metallophosphoesterase, which is reported as a component of nature biofilm and can help the resistance of biofilm to metal toxicity stronger than that of single cell (Cai et al. 2020). Gene *ypeQ* in Ph03 encoded a zinc-finger domain-containing protein. The most characteristic plant A20/AN1 zinc-finger domain protein is OSISAP1, which encoding gene is found to be responsive to different types of stresses that include heavy metals, cold, desiccation, etc. (Vij and Tyagi 2008). Gene *fnr* in Ph03 encoded a ferredoxin–NADP reductase. Zeng et al. (2014) find that a strategy for rice to cope with Cr toxicity is to activate antioxidant defense to mitigate Cr-induced oxidative stress. The antioxidant included ferredoxin–NADP reductase and NADP–isocitrate dehydrogenase, etc. (Zeng et al. 2014). Many metal efflux pumps are ABC transporter ATP-binding proteins (Chandrangsu et al. 2017). One gene encoding ABC transporter ATP-binding protein was detected in Ph05 of strain Bam1. Although more DNA islands (including GIs and Phs) are predicted in strain FZB42, only two genes involved in heavy metal resistance are predicted in its GIs and Phs. These two genes encode ABC transporter and arsenate efflux pump (Chen et al. 2007).

**Table 2:** Genes involved in heavy metal resistance in the genomic islands (GIs) and prophages (Phs) of the *B. amyloliquefaciens* strain Bam1 and *B. velezensis* strain FZB42

Bam1			FZB42 (Chen et al. 2007)		
DNA island	Gene	Product or function	DNA island	Gene	Product or function
GI03	gene2332	Heavy metal transporter	GI14	–	ABC transporter
GI06	*chrS/ywrC*	Regulation of chromate exporter	Ph03	–	Arsenate efflux pump
GI06	*chrA2/ywrA*	Chromate exporter			
GI06	*chrA1/ywrB*	Chromate exporter			
Ph02	*spoIISB*	Anti-toxin			
Ph03	*ypeQ*	Zinc-finger domain-containing protein			
Ph03	*pphA*	Metallophosphoesterase			
Ph05	*fnr*	Ferredoxin–NADP reductase 2			
Ph05	Gene3326	ABC transporter ATP-binding protein			

### Genome annotation of strain Bam1

**Fig. 2:**
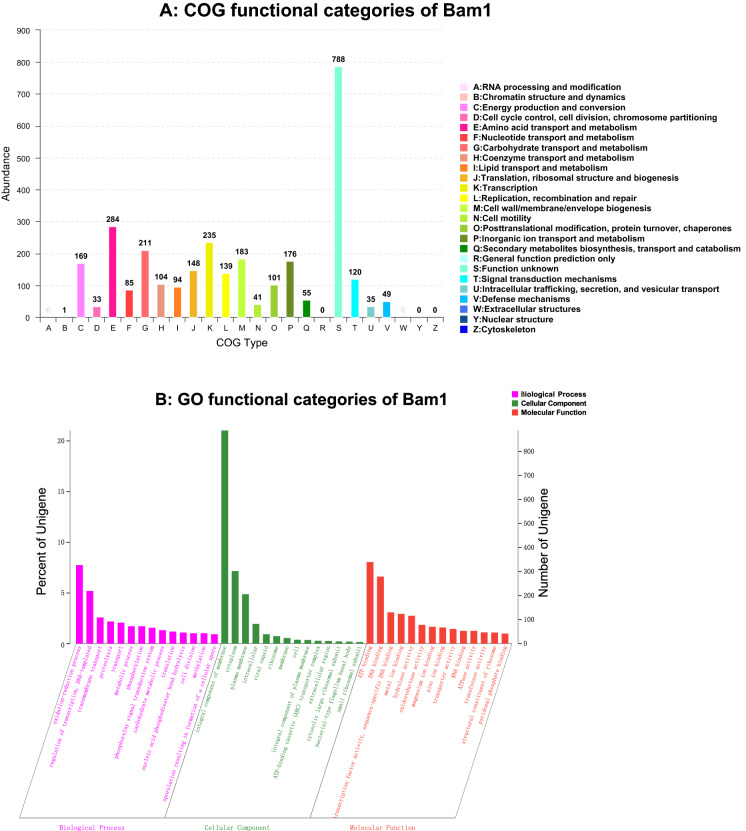
Distribution of genes across COG and GO functional categories in the chromosome of *B. amyloliquefaciens* strain Bam1

A total of 3003 genes of Bam1 were classified into 20 COGs (The Clusters of Orthologous Groups) families (Fig. 2A). The top five functional gene groups are involved in amino acid transport and metabolism, transcription, carbohydrate transport and metabolism, cell wall/membrane/envelope biogenesis, as well as inorganic ion transport and metabolism. The group of inorganic ion transport and metabolism contained 176 genes. The GO annotation showed that 3000 genes were classified into three big categories, biological process, cellular component, and molecular function, about 38 GO groups may involve in the heavy metal resistance in Bam1 (Fig. 2B, Additional file 1: Table S1). The KEGG orthologs were found for 2485 proteins by BLAST.

### Genome comparison within the ten *B. amyloliquefaciens* strains and* B. velezensis* strain FZB42

**Fig. 3:**
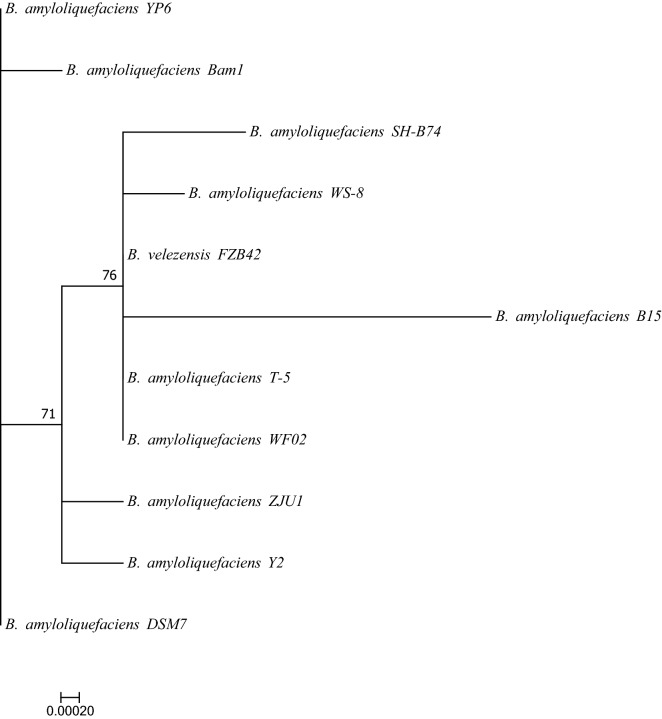
Phylogenetic tree for *B. amyloliquefaciens* strain Bam1, *B. velezensis* FZB42, and the nine other *B. amyloliquefaciens* strains based on homologous genes

**Fig. 4:**
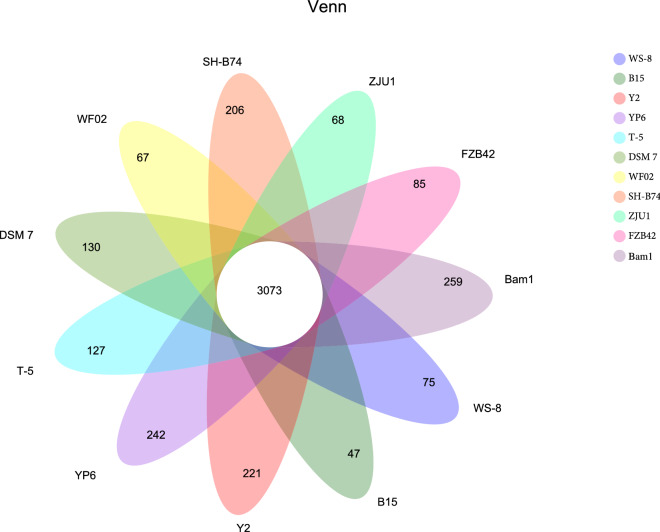
Venn diagram showing numbers of specific and shared gene families among the ten different *B. amyloliquefaciens* strains and *B. velezensis* FZB42

The phylogenetic tree (Fig. 3) and pan-genome analysis (Fig. 4) were all performed between strains Bam1, *B. velezensis* FZB42 (commercialized PGPR and biocontrol strain), and the nine other *B. amyloliquefaciens* strains. The nine other *B. amyloliquefaciens* strains were DSM7 (type strain), B15 (biocontrol strain), SH-B74 (biocontrol strain), T-5 (biocontrol strain), WF02 (biocontrol strain), WS-8 (biocontrol strain), ZJU1 (biocontrol strain), Y2 (PGPR strain) and YP6 (PGPR strain). The phylogenetic tree indicated that strain Bam1 was more close to strain DSM7 and YP6 than FZB42. While strain FZB42 is closer to *B. amyloliquefaciens* strains T-5 and WF02, which verified that *B. velezensis* is genetically very close to its ancestor *B. amyloliquefaciens*. The pan-genome analysis showed that 3073 gene families were found to be involved in the core genome shared by all of the eleven strains. The number of gene families unique to strain Bam1 was 259, which was the highest among the analyzed strains. While strain FZB42 possessed a relatively low number of unique gene families (85 gene families only).

### Genome comparison within the two *B. amyloliquefaciens* strains and *B. velezensis* strain FZB42

**Fig. 5:**
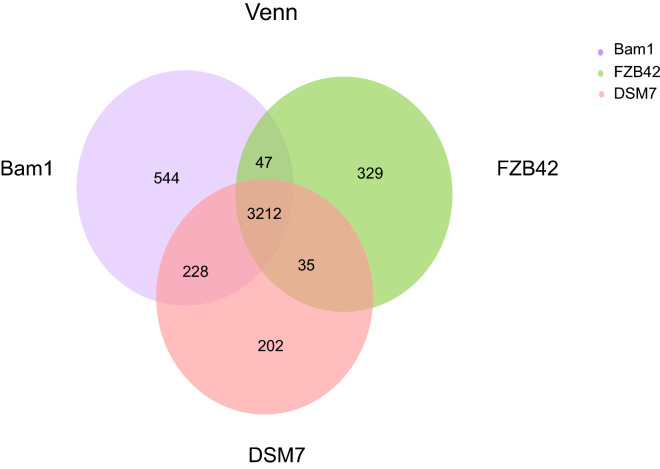
Venn diagram showing numbers of specific and shared gene families among the *B. amyloliquefaciens* strains of Bam1and DSM7, as well as *B. velezensis* FZB42

**Fig. 6:**
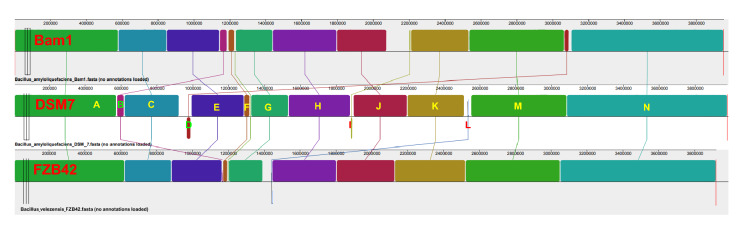
Co-linearity analysis for the *B. amyloliquefaciens* strains of Bam1 and DSM7, as well as *B. velezensis* FZB42

The pan-genome analysis (Fig. 5) and co-linearity analysis (Fig. 6) were performed between strain Bam1 and type strain DSM7, as well as the commercialized PGPR and biocontrol strain FZB42. The Venn diagram indicated that strain Bam1 still possessed the highest number of unique gene families (544 gene families) among the three strains. Some of the unique genes involved in the respiratory chain/electron transport chain, for example, the 6 genes from COG 2124 encoding Cytochrome P450 (Additional file 1: Table S2), which reported to affect heavy metal resistance in microbes and plants (Xu et al. 2015).

The three strains shared a relatively high co-linearity, because they possessed almost the same contigs in their genomes. However, still some differences between their genomes. The major differences included the very different arrangement of some contigs and the absence of some small contigs. Take contig B for example, the size of it in strain FZB42 was significantly smaller than those in strain Bam1 and DSM7. The arrangements of it in strain Bam1 and FZB42 were after contig E, while which was after contig A in strain DSM7. For contig D, it was arranged between contig C and E in strain DSM7, but between contig M and N in strain Bam1, and even missing in strain FZB42. For contig I, it was arranged after contig H in strain DSM7, while after contig J in strain Bam1, and missing in strain FZB42 also. For contig L, the arrangement of it in DSM7 was after contig K, while between contig G and H in FZB42, and absenting from strain Bam1.

### Comparison of genes involved in resistance to essential heavy metals between strains Bam1 and FZB42

The essential heavy metals of microorganisms include Mn, Fe, Zn, Cu, Co, etc. (Hooda 2010). They are very important for the growth of microbes, just a trace amount of them could maintain many life processes, but the excess of them could be toxic. To avoid the intoxication of metals, some fine systems had been found to maintain the metal homeostasis in different microbes (Chandrangsu et al. 2017). Compared with the non-essential heavy metals, there are more studies on the essential heavy metal homeostasis in bacteria. In general, metal limitation activates the pathways for import and mobilization of metals, whereas metal excess induces efflux and storage of metals in bacteria (Chandrangsu et al. 2017).

According to the previous studies (Huang et al. 2017a; Chandrangsu et al. 2017; Sachla et al. 2021), seventeen genes involved in manganese resistance were certainly found in the genome of strain Bam1, while just sixteen of them were found in the genome of strain FZB42 (Table 3). *rex*, encoding an NADH/NAD sensing repressor that controls the expression of cytochrome bd oxidase CydABCD (Sachla et al. 2021), was not found in strain FZB42. The highest and the lowest identifies of the other genes between the two strains were 98.70% and 93.76%, respectively. Among the seventeen genes, two genes (*mntH and citM, mntH* encoding an Mn-specific absorb pump) encoded Mn absorb proteins, *mntH* is the main Mn absorb pump in *B. subtilis* (Huang et al. 2017a). One gene (*mntR*) encoded a metalloregulator MntR, which is the central regulator of Mn^2+^ homeostasis. In *B. subtilis,* when intracellular Mn is limited, MntR can promote the absorption of Mn by derepressing the uptake system (MntH) (Huang et al. 2017a). When intracellular Mn is sufficient, it can repress the uptake system (MntH) to slow or stop the absorption of Mn (Huang et al. 2017a). When intracellular Mn is in excess, it can activate the efflux system (MneP) to export Mn (Huang et al. 2017a). Ten genes (*rex*, *qoxABCD*, *ctaAB*, *cydAB*, *mhqR*) were involved in the respiratory chain/electron transport chain (ETC) (Sachla et al. 2021). The deletion of *rex*, *qoxA*, *mhqR*, *ctaA*, and *cydA* in efflux pump lacking mutant is beneficial to the Mn resistance in *B. subtilis* (Sachla et al. 2021). One gene (*sodA*) encoded Mn-specific superoxide dismutase, which is reported to be the major pool of Mn^2+^ in the stationary phase (Chandrangsu et al. 2017). One gene (*opuD*) encoded a glycine betaine transporter, whose increased expression can alleviate the intoxication of excess Mn to the cell in *B. subtilis* (Sachla et al. 2021).

**Table 3:** Genes involved in Mn resistance in *B. amyloliquefaciens* strain Bam1 and *B. velezensis* strain FZB42

Gene	Location		Product or function	Identity of FZB24 (%)
	Bam1	FZB24		
*mntR*	2,427,694–2,428,122	2,416,837–2,417,265	Mn transport regulator	98.37
*mntH*	445,942–447,216	464,983–466,257	Mn transport protein; Mn^2+^ absorb pump	95.06
*citM*	733,033–734,331	773,138–774,436	Mg^2+^/citrate complex secondary transporter; uptake of citrate and Mg^2+^, Co^2+^, Ni^2+^, Mn^2+^	94.00
*mneP/ydfM*	249,478–250,371	258,470–259,363	Mn^2+^ efflux pump; Mn^2+^ export	98.43
*rex*	573,425–574,072	–	NADH/NAD sensing repressor that controls expression of cytochrome bd oxidase CydABCD; transcriptional repressor of anaerobically expressed genes involved in anaerobic respiration and fermentation	–
*cydA*	2,886,171–2,887,565	2,908,508–2,909,904	Cytochromed ubiquinol oxidase subunit I	95.42
*cydB*	2,887,588–2,888,628	3,703,711–3,704,727	Cytochromed ubiquinol oxidase subunit II	93.76
*ctaA*	1,466,975–1,467,901	1,474,483–1,475,409	Heme A synthase; heme biosynthesis	95.47
*ctaB/cyoE*	1,468,245–1,469,162	1,475,753–1,476,670	Protoheme IX farnesyltransferase 2; heme O synthase; heme biosynthesis; Converts heme B (protoheme IX) to heme O	97.39
*qoxA*	3,688,218–3,689,174	3,647,521–3,648,486	Cytochrome aa3-600 menaquinol oxidase subunit II	98.01
*qoxB*	3,686,241–3,688,190	3,645,544–3,647,493	Cytochrome aa3-600 menaquinol oxidase subunit I	98.21
*qoxC*	3,685,612–3,686,226	3,644,915–3,645,529	Cytochrome aa3-600 menaquinol oxidase subunit III	98.70
*qoxD*	3,685,242–3,685,610	3,644,544–3,644,913	Cytochrome aa3-600 menaquinol oxidase subunit IV	98.37
*mhqR*	1,356,332–1,356,778	1,305,481–1,305,927	MarR family transcriptional regulator, 2-MHQ and catechol-resistance regulon repressor	96.42
*sodA*	2,465,696–2,466,301	2,456,118–2,456,723	Fe/Mn superoxide dismutase; a major pool of Mn^2+^ in stationary phase	96.53
*mgtE*	1,324,228–1,325,583	1,275,422–1,276,777	Primary Mg transporter; Mg uptake; increased expression help to alleviate Mn toxicity	95.28
*opuD*	2,839,853–2,841,391	2,841,872–2,843,404	Glycine betaine transporter; increased expression can alleviate the Mn toxicity	94.98

Twenty-one genes related to iron resistance were predicted in strain Bam1, while one of them, *efeU*, encoding a high-affinity ferric iron (Fe^3+^) uptake protein, was absent in strain FZB42 (Table 4). The highest and lowest identities of the same genes between strain Bam1 and ZFB42 were 98.00% and 86.05%. Among the twenty-one genes, two genes (*fur*, *perR*) encoded the Fur family transcription regulator, Fur and PerR. Fur is a critical metalloregulator in Fe homeostasis, which is originally reported as a classic iron uptake regulator, sensing to Fe sufficient, but it is also reported as an efflux activator later (Guan et al. 2015; Chandrangsu et al. 2017). PerR is a paralog of Fur sensing to peroxide stress, which regulates putative metal transport and storage functions in *B. subtilis* (Moore and Helmann 2005). PerR has been reported sensing to Fe and Mn, but it is more sensitive to peroxides by virtue of oxidation reactions, which are enabled by the Fe corepressor in *B. subtilis* (Moore and Helmann 2005). Seventeen genes (*efe BOU*, *feuABC*, *fhuBCDG*, *yxeB*, *fpbNOPQ*, *fecC* and *yhfQ*) were involved in Fe uptake and acquisition, and all of them were repressed by Fur under Fe excess (Pi and Helmann 2017). Two genes (*fetB* and *pfeT*) encoded Fe efflux pumps. *pfeT* is activated by Fur and repressed by PerR in *B. subtilis* (Guan et al. 2015; Pi and Helmann 2017). However, *fetB* is not directly regulated by Fur, and its regulator is not yet known (Nicolaou et al. 2013). The increasing expression of *pfeT and fetB* help to improve the cell’s Fe resistance (Nicolaou et al. 2013; Pi and Helmann 2017).

**Table 4:** Genes involved in Fe resistance in *B. amyloliquefaciens* strain Bam1 and *B. velezensis* strain FZB42

Gene	Location		Product or function	Identity of FZB42 (%)
	Bam1	FZB24		
*fur*	2,334,984–2,335,433	2,252,893–2,253,342	Fur family transcriptional regulator of iron homoeostasis; sensor of Fe sufficiency; Fe uptake and efflux system regulator	98.00
*perR*	839,146–839,583	874,037–874,474	Fur family transcriptional regulator; peroxide stress response regulator; iron storage	96.35
*efeB*	3,696,580–3,697,836	3,655,884–3,657,140	Deferrochelatase/peroxidase EfeB; converts ferrous iron (Fe^2+^) to ferric iron (Fe^3+^) for uptake by EfeO–EfeU, peroxide detoxification under microaerobic conditions; heme peroxidase in elemental iron uptake;	93.95
*efeO*	3,697,856–3,699,007	3,657,159–3,657,395	Elemental iron uptake system (binding protein); high affinity uptake of ferric iron (Fe^3+^);	91.18
*efeU*	3,699,004–3,700,452	–	Elemental iron uptake system (permease); high-affinity iron transporter; ferric iron (Fe^3+^) uptake protein	–
*feuA*	182,176–183,132	187,111–188,067	ABC transporter for the siderophores Fe–enterobactin and Fe–bacillibactin (binding protein); acquisition of iron	96.13
*feuB*	181,149–182,156	186,084–187,091	ABC transporter for the siderophores Fe–enterobactin and Fe–bacillibactin (integral membrane protein); iron complex transport system permease protein; acquisition of iron	96.92
*feuC*	180,137–181,156	185,072–186,091	ABC transporter for the siderophores Fe–enterobactin and Fe–bacillibactin (integral membrane protein); iron complex transport system permease protein; acquisition of iron	93.33
*fhuB*	3,208,964–3,210,028	3,155,718–3,156,782	Hydroxamate siderophore ABC transporter (ATP-binding protein) (ferrichrome and ferrioxamine); siderophore uptake	94.37
*fhuC*	3,207,132–3,207,935	3,153,886–3,154,689	Hydroxamate siderophore ABC transporter (ATP-binding protein) (ferrichrome and ferrioxamine); siderophore uptake	94.53
*fhuD*	3,210,250–3,211,188	3,157,003–3,157,941	Hydroxamate siderophore ABC transporter (ATP-binding protein) (ferrichrome und ferrioxamine); siderophore uptake	95.53
*fhuG*	3,207,954–3,208,964	3,154,709–3,155,718	Hydroxamate siderophore ABC transporter (ATP-binding protein) (ferrichrome und ferrioxamine); siderophore uptake	93.37
*yxeB*	3,812,989–3,813,951	3,762,505–3,763,467	Hydroxamate siderophore ABC transporter; siderophore uptake	93.57
*fpbN/yclN*	384,472–385,425	403,591–404,544	Petrobactin ABC transporter (permease); acquisition of iron	94.97
*fpbO/yclO*	385,415–386,362	404,534–405,481	Petrobactin ABC transporter (permease); acquisition of iron	96.41
*fpbP/yclP*	386,356–387,114	405,475–406,233	Petrobactin ABC transporter (ATP-binding protein); acquisition of iron	95.13
*fpbQ/yclQ*	387,136–388,086	406,255–407,199	Petrobactin ABC transporter (binding protein); acquisition of iron	95.90
*fecC/yvrB*	3,197,604–3,198,650	3,144,353–3,145,399	Iron/citrate ABC transporter (binding protein); iron uptake	95.03
*yhfQ*	1,003,083–1,003,943	1,032,074–1,032,952	Iron/citrate ABC transporter (solute-binding protein); iron uptake	86.05
*fetB/yjkA*	1,231,201–1,231,962	1,202,052–1,202,813	Iron export ABC transporter permease subunit FetB; iron export	93.44
*pfeT/zntA/zosA*	1,370,249–1,372,162	1,321,623–1,323,536	Fe^2+^/Zn^2+^/Cd^2+^-exporting ATPase; cation efflux	91.54

Eight genes were found in strain Bam1 and FZB42 involved in zinc resistance (Table 5), including two regulator encoding genes (*zurR* and *czrA*), three Zn uptake protein-encoding genes (*znu*ABC), and three export protein-encoding genes (*cadA*, *czcD*, and *pfeT*). Among these genes, the highest identity between the two strains was 97.69% of *czrA*, and the lowest identity was 89.40% of *czcD*. ZurR is also a paralog of Fur, a Zn-specific metalloregulatory protein, involves in the regulation of the Zn uptake system (Chandrangsu et al. 2017). The uptake system encoded by *znuABC* is specifically responded to Zn and is repressed by ZurR under Zn excess condition (Moore and Helmann 2005). However, the efflux systems are not responded to Zn specifically and are also not regulated by ZurR. Zn could be pump out of cells by *cadA*, *czcD* and *pfeT* encoding proteins when Zn was excess according to the genomes of strain Bam1 and FZB42*. pfeT* is regulated by both Fur and PerR aforementioned, as well as can export cations, such as Zn^2+^, Fe^2+^, and Cd^2+^, out of cells when they are excess (Moore and Helmann 2005). *cadA* and *czcD* are regulated by CzrA. CzrA is an ArsR family regulator, can dissociate from DNA upon metallation with Zn^2+^, and then results in the induction of two efflux systems, the CadA P-type ATPase and the CzcD cation diffusion facilitator type transporter (Chandrangsu et al. 2017). CzcD can export not only Zn^2+^, but also Co^2+^, Cu^2+^, Cd^2+^ and Ni^2+^ (Gaballa et al. 2012). CadA mainly functions as a Cd efflux pump and is also reported as a non-specific Zn efflux pump (Gaballa and Helmann 2003).

**Table 5:** Genes involved in Zn, Cu and Co resistance in *B. amyloliquefaciens* strain Bam1 and *B. velezensis* strain FZB42

Metal	Gene	Location		Product or function	Identity of FZB42 (%)
		Bam1	FZB24		
Zn	*zurR*	2,470,670–2,471,080	2,461,096–2,461,506	Zn-specific metalloregulatory protein; Fur family transcriptional regulator; Zn uptake regulator	97.32
	*znuA*	275,215–276,174	283,478–284,437	Zn transport system substrate-binding protein; Zn-uptake complex component A periplasmic	95.94
	*znuB*	276,882–277,721	285,145–285,984	ABC transporter for Zn (permease); Zn uptake	96.43
	*znuC*	276,229–276,924	284,492–285,187	Zn ABC transporter ATP-binding protein; Zn uptake	96.98
	*czrA*	1,940,021–1,940,323	2,011,551–2,011,933	ArsR family transcriptional repressor, regulation of resistance against toxic metal cations; regulating the expression of *cadA*, *czcD*	97.69
	*cadA*	3,237,502–3,239,613	3,183,406–3,185,517	Cd-translocating P-type ATPase; Cd^2+^/Zn^2+^/Co^2+^ efflux	93.51
	*czcD*	506,576–507,511	537,946–538,876	Cation exporter (antiporter); cation efflux; resistance against Zn^2+^, Cu^2+^, Co^2+^, Ni^2+^, Cd^2+^	89.40
	*pfeT/zntA/zosA*	1,370,249–1,372,162	1,321,623–1,323,536	Fe^2+^/Zn^2+^/Cd^2+^-exporting ATPase; cation efflux	91.54
Cu	*ycnK*	400,613–401,185	407,200–407,772	DeoR family transcription repressor; copper-responsive transcription repressor of the *ycnK-ycnJ* operon; regulation of copper uptake	97.73
	*ycnJ*	398,952–400,580	418,065–419,693	Copper transport protein YcnJ; uptake of copper	93.99
	*csoR*	3,242,556–3,242,855	3,188,475–3,188,767	Copper-sensing transcriptional repressor CsoR; control of copper homeostasis, including copper import and export	96.25
	*copZ*	3,242,279–3,242,485	3,188,180–3,188,386	Copper chaperone CopZ; copper transport protein; resistance to copper;	95.17
	*copA*	3,239,760–3,242,195	3,185,667–3,188,096	Copper-exporting P-type ATPase A; copper export	92.90
	*czcD*	506,576–507,511	537,946–538,876	Cation exporter (antiporter); cation efflux; resistance against Zn^2+^, Cu^2+^, Co^2+^, Ni^2+^, Cd^2+^	89.40
Co	*ecfA1/ybxA/thiW*	1,319,552–1,321,189	1,270,763–1,272,400	Cobalt ABC transporter (ATP-binding protein); uptake of micronutrients	90.36
	*ecfT/ybaF*	152,693–153,490	152,878–153,675	Cobalt ABC transporter (permease); uptake of micronutrients	94.36
	*citM*	733,033–734,331	773,138–774,436	Mg^2+^/citrate complex secondary transporter; uptake of citrate and Mg^2+^, Co^2+^, Ni^2+^, Mn^2+^	94.00
	*cadA*	3,237,502–3,239,613	3,183,406–3,185,517	Cd-translocating P-type ATPase; Cd^2+^/Zn^2+^/Co^2+^ efflux	93.51
	*czcD*	506,576–507,511	537,946–538,876	Cation exporter (antiporter); cation efflux; resistance against Zn^2+^, Cu^2+^, Co^2+^, Ni^2+^, Cd^2+^	89.40

Six genes involved with Copper resistance were predicted both in strain Bam1 and FZB42, including two regulators encoding genes (*ycnK* and *csoR*), one import protein encoding gene (*ycnJ*), one copper chaperone encoding gene (*copZ*), and two export encoding genes (*copA* and *czcD*) (Table 5). The identity of *ycnK* was the highest (97.73%) between strain Bam1 and FZB42, while the identity of *czcD* was the lowest (89.40%). YcnK is a Cu-specific regulator that represses the expression of the Cu-specific uptake system, YcnJ, under Cu excess conditions (Chillappagari et al. 2009). CsoR is a Cu-responsive MerR homolog, classically reported to regulate the *copZA* operon, which encodes a Cu chaperone and a Cu P-type ATPase for Cu export, respectively (Moore and Helmann 2005). CsoR will derepress the *copZA* operon and *ycnJ* under Cu excess condition (Chillappagari et al. 2009). CzcD is also reported as a non-specific efflux pump of Cu (Chillappagari et al. 2009; Gaballa et al. 2012).

Cobalt is just required by some atmospheric–nitrogen fixation bacteria (Hooda 2010), and the synthesis pathway for cobalamin is lacking in many *Bacillus* (except *B. megaterium*) (Moore and Helmann 2005). Five genes related to cobalt resistance were still detected in both strain Bam1 and FZB42 (Table 5). Among these five genes, three of them (*ecfA1*, *ecfT*, and *citM*) encoded the cobalt uptake system, and two of them (*czcD* and *cadA*) encoded the cobalt efflux system. *ecfA1*and *ecfT* encoded cobalt ABC transporters, which also have a Fe uptake function. CitM is a non-specific cation uptake protein, which is reported could help to uptake cobalt. CzcD and CadA are reported as non-specific cation efflux pumps of cobalt (Moore and Helmann 2005). The homology of cobalt resistance-related genes was relatively lower than that of other heavy metal resistance-related genes, and the highest identity was only 94.36%. The regulator of Co uptake protein-encoding gene, *ecfA1 and ecfT*, is not yet known.

### Comparison of genes involved in resistance of non-essential heavy metals between strains Bam1 and FZB42

When applied on cultivated land, the biocontrol or PGPR strains inevitably encountered metal excess stress. Because many agricultural soils, especially in Southeast Asia, have been lightly contaminated by heavy metals for the frequent application of chemical pesticides and chemical fertilizers, as well as the rapid development of the industry (Hooda 2010). The reported non-essential heavy metals (or metalloids) detected in agricultural soils included Cd, Cr, As, Pb, and Hg (Hooda 2010). Compared with the essential heavy metals, little is known about the resistance mechanism of *Bacillus* to non-essential heavy metals.

Cadmium is the primary pollutant in cultivated land, especially in China (Hooda 2010; Huang et al. 2017b). Six genes involved in Cd resistance were found in the genomes of strain Bam1 and FZB42 (Table 6). The products of two genes (*czrA*, *arsR*) acted as the regulators of different efflux systems. The rest genes (*cadA*, *czcD*, *pfeT*, and *arsB*) encoded four efflux pumps. The identities of these genes between the two strains were from 97.69 to 89.40%. *czrA* encodes CzrA, which is an ArsR/SmtB family repressor that binds to the *cadA* and *czcD* regulatory regions and is released upon interaction with metal ions (such as Cd, Zn, Co, etc.) (Moore et al. 2005). CadA is a P-type ATPase that mainly effluxes Cd^2+^ in *B. subtilis* (Gaballa and Helmann 2003). CzcD and PfeT are non-specific Cd efflux systems (Moore and Helmann 2005). The mechanism by which the *arsR-arsB* operon contributes to Cd resistance is not yet clear, though they help to mitigate the Cd intoxication in *B. subtilis* (Moore et al. 2005).

**Table 6:** Genes involved in Cd, Cr, As and Pb resistance in *B. amyloliquefaciens* strain Bam1 and *B. velezensis* strain FZB42

Metal	Gene	Location		Product or function	Identity of FZB42 (%)
		Bam1	FZB24		
Cd	*czrA*	1,940,021–1,940,323	2,011,551–2,011,933	ArsR family transcriptional repressor, regulation of resistance against toxic metal cations; regulating the expression of *cadA*, *czcD*	97.69
	*cadA*	3,237,502–3,239,613	3,183,406–3,185,517	Cd-translocating P-type ATPase; Cd^2+^/Zn^2+^/Co^2+^ efflux	93.51
	*czcD*	506,576–507,511	537,946–538,876	Cation exporter (antiporter); cation efflux; resistance against Zn^2+^, Cu^2+^, Co^2+^, Ni^2+^, Cd^2+^	89.40
	*pfeT/zntA/zosA*	1,370,249–1,372,162	1,321,623–1,323,536	Fe^2+^/Zn^2+^/Cd^2+^/Pb^2+^-exporting ATPase; cation efflux	91.54
	*arsR*	1,940,021–1,940,323	1,978,959–1,979,261	ArsR family transcriptional regulator; regulation the expression of *arsB*, may have the potential to sequester Cd	97.69
	*arsB*	3,494,481–3,495,812	3,687,147–3,688,445	Arsenical pump membrane protein; arsenite exporter, may efflux Cd^2+^	93.32
Cr	*chrS/ywrC*	3,502,399–3,502,875	3,450,892–3,451,368	AsnC family transcriptional repressor; regulation of chromate export	94.55
	*chrA1/ywrB*	3,502,889–3,503,479	3,451,382–3,451,972	Chromate transporter ChrA1/YwrB; chromate exporter; chromate detoxification	89.00
	*chrA2/ywrA*	3,503,497–3,504,012	3,451,990–3,452,505	Chromate transporter ChrA2/YwrA; chromate exporter; chromate detoxification	92.64
As	*arsR*	1,940,021–1,940,323	1,978,959–1,979,261	ArsR family transcriptional regulator; regulation the expression of *arsB* and *arsC*	97.69
	*arsB*	3,494,481–3,495,812	3,687,147–3,688,445	As^3+^ pump membrane protein; As^3+^ export	93.32
	*arsC*	3,160,891–3,161,247	3,105,252–3,105,608	As^5+^ reductase	96.64
	*aseR*	3,729,932–3,730,282	3,686,783–3,687,133	ArsR family transcriptional repressor; regulation of As^3+^ efflux pump ArsA	89.74
	*arsA/ydfA/yqcL*	3,730,296–3,731,594	3,687,147–3,688,439	As^3+^ pump membrane protein; As^3+^ efflux pump; detoxification of As^3+^ and As^5+^	93.35
Pb	*cadA*	3,237,502–3,239,613	3,183,406–3,185,517	Cd-translocating P-type ATPase; Cd^2+^/Zn^2+^/Co^2+^ efflux; Pb^2+^ efflux in *S. aureus*	93.51
	*pfeT/zntA/zosA*	1,370,249–1,372,162	1,321,623–1,323,536	Fe^2+^/Zn^2+^/Cd^2+^-exporting ATPase; cation efflux; Pb^2+^ efflux in *E. coli*	91.54

Chromium is another important pollutant in cultivated land (Hooda 2010). Three genes were predicted related to the Cr resistance in strain Bam1 and FZB42, and the identities of the three genes were from 94.55 to 89.00% (Table 6). *chrS* encode an Lrp/AsnC family Cr-specific regulator ChrS, which negatively regulated the expression of *chrA* and *chrB*, two Cr-specific efflux pump encoding genes (Aguilar-Barajas et al. 2013; He et al. 2018). When Cr is excess, Cr ions will remove the negative effect of ChrS on ChrA and ChrB, then ChrA and ChrB will export the Cr out of the cell to alleviate the Cr toxicity of *B. subtilis* (Aguilar-Barajas et al. 2013).

Arsenic is a kind of metalloid detected in agricultural soils, which can cause symptoms similar to that of heavy metal intoxication (Hooda 2010). Five genes involved in As resistance were predicted in strain Bam1 and FZB42 (Table 6). The highest and lowest identities of these genes, were 97.69% (*arsR*) and 89.74% (*aseR*), respectively, belonging to two regulator encoding genes. ArsR, an ArsR/SmtB family metalloregulators, negatively regulates itself (*arsR*) and genes encoding an As^3+^ efflux pump (*arsB*) and an As^5+^ reductase (*arsC*) (Moore et al. 2005). Another As^3+^ sensing regulator, AseR, which is also a member of the ArsR family, regulates an ArsB homolog protein, ArsA, another As^3+^ efflux pump (Moore et al. 2005). In these systems, the As^5+^ will be reduced into As^3+^ by ArsC, then As^3+^ will be exported by the efflux, ArsB, or ArsA (Moore et al. 2005). It means that the *ars* operon encodes both an arsenate reductase and an arsenite efflux pump, and is required for As^5+^ resistance, while the *aseRA* operon mainly contributes to As^3+^, but not As^5+^, resistance (Moore et al. 2005).

Lead and mercury resistance systems are poorly characterized in *Bacillus*. Only two Pb non-specific efflux pumps encoding genes (*cadA* and *pfeT*) were detected in strain Bam1 and FZB42 (Table 6). While there were not any genes related to Hg resistance detected in these two strains.

### The resistance of strains Bam1 and FZB42 to different heavy metals

**Fig. 7:**
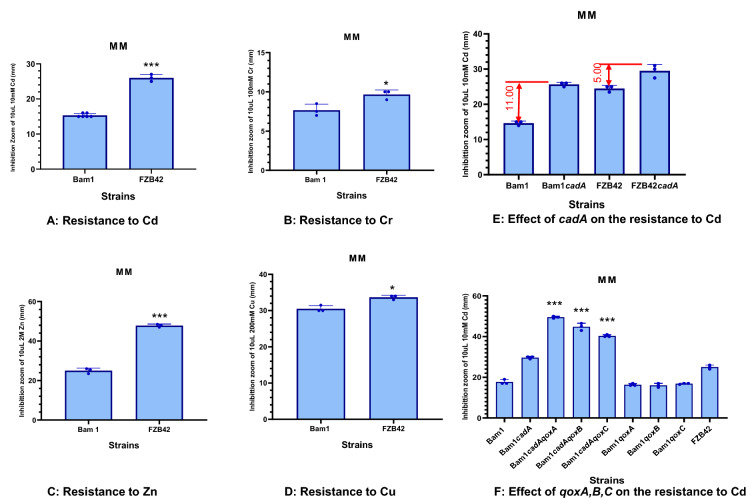
Resistances of *B. amyloliquefaciens* strain Bam1, *B. velezensis* FZB42 and their mutants to different heavy metals. All measurements are mean ± SEM (*n* = 3). *** represents significant difference (*P* < 0.01), * represents significant difference (*P* < 0.05)

**Fig. 8:**
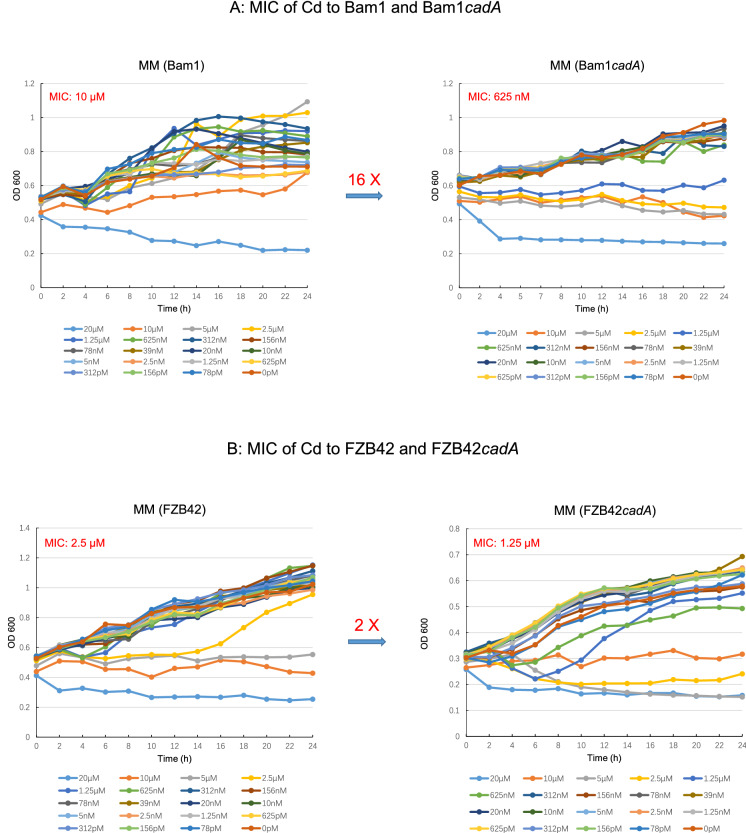
MIC of Cd to *B. amyloliquefaciens* strain Bam1 and *B. velezensis* FZB42 as well as their *cadA* deletion mutants. All measurements are mean ± SEM (*n* = 3)

The resistance of strain Bam1 and FZB42 to four kinds of heavy metals was evaluated by disk diffusion assay and MIC assay. These four kinds of heavy metals included non-essential heavy metals, Cd and Cr, as well as essential heavy metals, Zn and Cu, which were the major agricultural soil polluting heavy metals in China.^8^ The results showed that the resistance of stain Bam1 to these four kinds of heavy metals were significantly better than those of strain FZB42 (Figs. 7, 8 and Additional file 1: Fig. S3). The differences in the resistance of these two stains to Cd and Zn were significant at 0.01 level (Fig. 7A, C), while the differences in the resistance of these two stains to Cr and Cu were only significant at 0.05 level (Fig. 7B, D). The results of MIC assay showed a similar trend as those in disk diffusion assay. The MIC of Cd to strain FZB42 was 4 times that to strain Bam1 (Fig. 8A, B). The MIC of the rest three heavy metals (Cr, Zn, and Cu) to strain FZB42 were all 2 times as those to strain Bam1 (Additional file 1: Fig. S3).

### The resistance of *cadA* and *qox* deletion mutants of strains Bam1 and FZB42 to cadmium

CadA is reported as the key factor (key efflux pump of Cd) affecting Cd resistance in *B. subtilis* (Gaballa and Helmann 2003). The homology of *cadA* gene in *B. amyloliquefaciens* strain Bam1 and FZB42 was very high (the identity between them reached 93.51%). Therefore, we wondered whether there was any difference in the effect of *cadA* on the Cd efflux ability of these two strains. Then the *cadA* deletion mutants of these two strains (Bam1*cadA*, FZB42*cadA*) were constructed and the evaluation of Cd resistance was performed between them and their wild-type strains. The results indicated that the Cd efflux ability affected by *cadA* in strain Bam1 was much higher than that in strain FZB42. When *cadA* was deleted from strain Bam1 the diameter of the inhibition zoom expanded by 11.00 mm, while in strain FZB42 only expanded by 5.00 mm (Fig. 7E). This trend was exhibited again in the MIC experiment, the *cadA* deletion resulted in a 16 times decrease of MIC in strain Bam1 (Fig. 8A), while just 2 times in strain FZB42 (Fig. 8B). Although the difference of *cadA* between strain Bam1 and FZB42 was small, this small difference still made the two strains possess distinct resistance to Cd.

Except for the traditional Cd efflux pump, CadA, we also found that the encoding genes of an important terminal oxidase in the respiratory chain, Qox, greatly affected the Cd resistance of the Bam1 strain under Cd stress (Fig. 7F). When *qoxA*, *qoxB*, and *qoxC* were deleted from the *cadA* lacking strain (Bam1*cadA*), the Cd resistance of these mutants decreased significantly compared with Bam1*cadA* and Bam1. However, when they were only deleted from the WT strain (Bam1), the Cd resistance was similar to that of WT.

## Discussion

Metals cannot be synthesized or degraded; therefore, their homeostasis primarily relies on modulating transport into and out of the cells (Chandrangsu et al. 2017). In general, metal excess typically leads to the repression of the import system and the activation of the efflux systems in bacteria (Chandrangsu et al. 2017; Huang et al. 2017b). In *Bacillus*, the studies of resistance to essential trace elements are much more detailed than those of non-essential elements (Moore and Helmann 2005; Sachla et al. 2021). Similar results appeared in our study. For the homeostasis systems of essential trace elements in *B. amyloliquefaciens* strain Bam1 and *B. velezensis* strain FZB42, except that only the import and export systems of Co were detected, while no specific regulator sensing to Co was found; the corresponding regulators, as well as import and export systems of other elements (Mn, Fe, Zn, and Cu), were all detected. Take the homeostasis systems of iron as an example, since iron is the most needed trace element of most organisms, only in the absorption system, more than a dozen related genes were found in the genomes of the two strains (17 genes in the genome of strain Bam1 and 16 genes in the genome of FZB42). The iron absorption system included the uptake and acquisition proteins of element iron (*efeBOU*), ferric–bacillibactin and ferric–enterobactin (*feuABC*), ferric–petrobactin (*fpbNOPQ*), ferric citrate (*fecC*, *yhfQ*), as well as hydroxamate siderophores (*fhuBCGD*) (Table 4). All of these genes will be repressed by Fur when Fe is in excess (Moore and Helmann 2005). Because the non-essential trace elements (Cd, Cr, As, and Pb) are not needed for the growth of microbes, in the homeostasis systems of these two strains, only some specific and non-specific efflux pumps but no uptake proteins were detected. The specific efflux pump regulators were just detected for Cd, Cr, and As, but not for Pb.

Concerning the homeostasis systems of essential trace elements, some regulators are bifunctional. For example, MntR is a classical bifunctional metalloprotein, which is reported as an Mn^2+^-activated transcription factor, will repress the expression of the uptake system (MntH and MntABCD) when Mn is sufficient and will derepress the expression of the efflux system (MneP and MneS) when Mn is excess in *B. subtilis* (Huang et al. 2017b). Fur is another bifunctional regulator sensing Fe, which was reported as a typical regulator of the Fe uptake system previously (Ma et al. 2012), while it also has been reported could regulate the Fe efflux in *B. subtilis* by inducing the expression of PfeT directly under conditions of Fe^2+^ intoxication lately (Guan et al. 2015; Chandrangsu et al. 2017). CsoR is also reported to possess bifunction in the regulation of copper homeostasis systems in *B. subtilis* (Chillappagari et al. 2009). CsoR is originally reported regulated *copZA* copper efflux system, within which the copper chaperone, CopZ, can bind Cu^+^_,_ and then transfer it to the copper exporter, CopA, for efflux (Banci and Rosato 2003; Moore and Helmann 2005). While lately CsoR was also reported negatively regulated the Cu uptake system, YcnJ, which is reported majorly regulated by YcnK (Chillappagari et al. 2009). Under the Cu excess condition, CsoR derepresses the expression of *copZA* operon and *ycnJ*, while the expression of *ycnJ* is also strongly repressed by YcnK in this condition, then the combined effect helps to improve the cell’s Cu resistance (Chillappagari et al. 2009).

Except for the regulators with respect to the homeostasis systems of Mn, Fe, and Cu were bifunctional, the other regulators involved in the homeostasis systems of essential trace elements were monofunctional, which just regulate the uptake system or efflux system in these two strains. Take zinc homeostasis systems, for example, regulator ZurR regulates the Zn-specific uptake system ZnuABC, while CzrA regulates two non-Zn-specific efflux pumps, CadA and CzcD, which help to alleviate the cell’s Zn^2+^ intoxication under Zn excess condition.

For the homeostasis systems of non-essential trace elements (Cd, Cr, As, Pb, and Hg) in strain Bam1 and FZB42, no specific uptake systems and corresponding regulators were detected, because these elements are generally not needed for the growth of microbes and can lead to intoxication with very low concentration. Therefore, some genes encoding specific efflux pumps and their corresponding regulators, as well as non-specific efflux pumps, were mainly detected in the genomes of the two strains.

For the Cd export systems in *B. subtilis*, CadA is reported as the major determinant for Cd^2+^ resistance, which is activated by CzrA under Cd excess conditions (Moore et al. 2005). Though CzrA is originally reported sensing to Zn and activated two efflux pumps, CadA and CzcD, for Zn and many other kinds of cation (including Cd, Cu, Co, etc.) efflux under metal ion excess (Moore and Helmann 2005). Whereas in our study, the role of CadA for Cd efflux is not very important in *B. subtilis* strain, but crucial in *B. amyloliquefaciens* strain Bam1 (Additional file 1: Fig. S4). Furthermore, the efficiency of *cadA* in strain Bam1 (with strong resistance to Cd) and strain FZB42 (with weak resistance to Cd) is very different (Figs. 7E and 8). Except for the CzrA–CadA/CzcD system, the ArsR–ArsB system is also reported could protect the cell against the elevated levels of Cd (Moore et al. 2005). The ArsR–ArsB system is originally reported as As export system, though the mechanism of it for Cd resistance is not yet clear, researchers infer that ArsB may efflux Cd^2+^ (Moore et al. 2005). For lead export, just two non-specific efflux pumps encoding genes, *cadA* and *pfeT* were detected in strain Bam1 and FZB42. Concerning these two efflux pumps, PfeT (named ZntA in *Escherichia coli*) is reported to export Pb in *E. coli* (Mitra and Sharma 2011), while CadA efflux Pb in *Staphylococcus aureus* (Tsai et al. 2002). Not any genes involved in the Hg export system were detected in *B. amyloliquefaciens* strains, including Bam1 and FZB42. The most extensively investigated Hg resistance mechanism is encoded by the *mer* operon genes, which are often localized on mobile genetic elements, such as plasmids and transposons (Matsui et al. 2016). Not any of the plasmids and transposons were predicted in strains Bam1 and FZB42, which might be the reason why no genes related to Hg export were detected in these two strains. Among the Hg resistant strains, the *mer* operon in the narrow-spectrum Hg resistant *Bacillus* strains (only having resistance to inorganic mercury salts, such as *B. cereus* strain TA32–5 and *B. licheniformis* strain FA6–12) includes *merRETPA*, whereas in the broad-spectrum Hg resistant *Bacillus* strains (having resistance to organic and inorganic mercury salts, such as *B. megaterium* strain MB1) include *merRETPA* and *merB* (Matsui et al. 2016). When under Hg stress, the protein conformation of MerR will be changed by attaching Hg^2+^, then activated the transcription of the other genes in this operon to help the cell against Hg intoxication. First, the organic Hg will be transferred into inorganic Hg^2+^ by MerB, an organomercury lyase. Secondary, a transporter, MerP, binds Hg^2+^, then transfers it to another transporter, MerT. Next, the Hg^2+^ is transferred from MerT to MerA (a mercury reductase), which can transform the Hg^2+^ into Hg^0^. Finally, the volatile Hg^0^ will be released into the air and alleviate the intoxication of the cell (Hamlett et al. 1992).

The importance of metal storage and sequestration mechanisms, as well as the metal resistance affected by the Electron transport chain (ETC) or respiratory chain (RC), is becoming increasingly appreciated except for import and export systems (Chandrangsu et al. 2017; Sachla et al. 2021). For Mn resistance, 10 genes (*rex*, *cydAB*, *ctaAB*, *qoxABCD*, *mhqR*) involved in ETC/RC were detected in strain Bam1, while only 9 of them were detected in strain FZB42 (lacking *rex*). The main function of these genes is to eliminate reactive radical species (RRS) and their derivatives caused by Mn stress (Sachla et al. 2021). SodA (Mn-dependent superoxide dismutase) is the major pool of Mn in *Bacillus*, which can store the excess Mn and eliminate the reactive oxygen species (ROS) caused by Mn-stress, then alleviate the Mn intoxication (Kim et al. 1998; Sachla et al. 2021). PerR is also reported to act as the regulator of iron storage in *B. subtilis* (Carrondo 2003; Guan et al. 2015). In Cd export system, ArsR may have the potential to sequester Cd except for regulating the expression of the efflux pump, ArsB, because it contains 6 cysteines (Cys), which may provide it the sequestration of Cd (Moore et al. 2005).

For the important aa3-type terminal oxidase in the respiratory chain, Qox, which is reported to improve the Mn resistance in *B. subtilis* when its encoding genes were deleted (Sachla et al. 2021). Whereas in this study, we found the role of Qox was opposite in Cd resistance of Bam1 to that in Mn resistance of *B. subtilis*. When the encoding genes of Qox were deleted from the Cd efflux pump lacking strain (Bam1*cadA*), the Cd resistance decreased sharply (Fig. 7F). To our knowledge, it was the first report that Qox could affect Cd resistance in microbe. The mechanisms of Qox affecting Cd and Mn resistance are seen to be different and need to be further studied.

Although there were not very high differences in the homology of heavy metal resistance-related genes between strains Bam1 and FZB42 (except for the lacking genes, the lowest identity among these genes was 86.05%), these small differences in the related genes could result in very different heavy metal resistances (Figs. 7A and 8). The differences in the heavy metal resistance-related genes coupled with other genomic differences led to significant differences in the heavy metal resistance of these two strains (Figs. 7, 8 and Additional file 1: Fig. S3). The other differences among the genomes included the different arrangement of the contigs and the numbers of the heavy metal resistance genes among the GIs and Phs. Contig is a large DNA fragment generated by overlapping series of sequence reads and contains many genes. Therefore, the lack of one or some contigs may lead to a decrease or loss of some function of an organism. For example, gene2322 (Heavy metal transporter, in GI03, from 2,161,727 to 2,164,792) and *pphA* (Metallophosphoesterase, in Ph03, from 2,110,424 to 2,109,708) were located in contig I of strain Bam1, while this contig lacks in strain FZB42. This might be one of the reasons that led to a different heavy metal resistance between strain Bam1 and FZB42. GI and Ph are the evolutionary results of bacteria to adapt to environmental changes or improve their survival competitiveness. There only twelve DNA islands were predicted in the genome of Bam1, while seventeen of those were predicted in the genome of FZB42 (Table 2). In strain Bam1, nine genes related to the resistance to the heavy metal were found in five DNA islands (GI03, GI06, Ph02, Ph03, Ph05), and the rest DNA islands were involved in symbiosis with phage, material transportation, senescent cell structures clearance, as well as gene regulation (Additional file 1: Table S3). Whereas in strain FZB42, only two genes related to the resistance to the heavy metal were found in two DNA islands (GI14, and Ph03), the functions of the rest fifteen islands included multidrug (or antibiotics) resistance, the remnants of phages, extracellular arabinogalactan hydrolysis, galactose uptake and catabolism, ester cyclization, non-ribosomal peptide synthesis, gene regulation, etc. (Chen et al. 2007). From the above illustration, we could conclude that more DNA islands of strain Bam1 were involved in heavy metal resistance, while fewer DNA islands of strain FZB42 were involved in this function. That might be the reason why strain Bam1 possessed a stronger ability to resist the heavy metal than strain FZB42. Therefore, it can be deduced that the variation of heavy metal resistance-related genes and the variation of genomes between strains FZB42 and Bam1 lead to significant differences in their resistance to heavy metals.

*Bacillus amyloliquefaciens* was originally classified as *B. subtilis*, then separated from it in 1967 for the ability to produce a large amount of α-amylase (Welker and Campbell 1967). The close relation made them have high similarities in genomes and some functions or mechanisms (including the mechanism of heavy metal resistance). For example, most of the genes related to heavy metal resistance in *B. subtilis* were also detected in *B. amyloliquefaciens*. However, due to the difference in their genomes, there are still some obvious differences in the mechanism of heavy metal resistance, which may lead to their differences in heavy metal resistance. The function of the above-mentioned CadA, a Cd major efflux pump, is very different between the two species. When *cadA* was deleted from *B. subtilis* strain CU1065 (the derivant of *B. subtilis* stander strain 168), which showed little effect on its Cd resistance, but when *cadA* was deleted from *B. amyloliquefaciens* strain Bam1, which significantly decreased the Cd resistance of strain Bam1 (Additional file 1: Fig. S4). The identity of *cadA* between *B. amyloliquefaciens* strain Bam1and *B. subtilis* strain 168 was 72.90%. It could be inferred that the variation of *cadA* leads to the contrary ability of Cd efflux between the two strains. For Cd resistance, besides the difference in *cadA*, there was still another difference in the mechanism in ArsR between these two spices, which may also result in their different Cd resistance. In *B. subtilis*, *ars* operon may not only sequester Cd^2+^ by ArsR, but also by protein YqcK (the product of ars operon with no known function) for its containing three pairs of consecutive Cys residues near its C-terminus (Moore et al. 2005). While in *B. amyloliquefaciens*, the encoding gene of YqcK was not detected. These results implied that *B. subtilis* might sequester Cd^2+^ by ArsR and YqcK, whereas *B. amyloliquefaciens* just sequester Cd^2+^ by ArsR. Therefore, when studying the mechanism of heavy metal resistance of *B. amyloliquefaciens*, we cannot just refer to its closely related species, *B. subtilis.*

PGPR or biocontrol strains inevitably face the stress of essential and non-essential heavy metals in their product development and application in the field. In this study, the resistance mechanism of biocontrol strain, *B. amyloliquefaciens* Bam1, to several common essential and non-essential heavy metals in agriculture was studied by methods of the comparative genomic analysis, which will provide a scientific basis for the development of compound biological fertilizers or microbial pesticides, as well as the application technology of the products. To our knowledge, this is the first study on the mechanism of the resistance of *B. amyloliquefaciens* to heavy metals, and it is also the first report on the mechanism of the resistance of the biocontrol agent to heavy metals.

## Conclusions

In summary, the resistance mechanisms of strain *B. amyloliquefaciens* Bam1 to the heavy metals could be deduced as follows:

For the essential heavy metals, Bam1 promoted its heavy metal resistance mainly by decreasing the import and increasing the export of heavy metal ions with the corresponding homeostasis systems, which are regulated by different metalloregulators. Under Mn stress, the bifunctional regulator, MntR, would repress the Mn uptake protein, MntH, and derepress the Mn efflux pump, MneP. While eliminating RRS by ETC/RC-related enzymes, and storing excess Mn through Mn-SOD were also the mechanisms by which Bam1 improved its Mn resistance. When Fe was excess, another bifunctional regulator, Fur, would repress the uptake proteins of various iron, then the Fe efflux pump, PfeT, would be activated by Fur and PerR; another Fe exporter, FetB, would also be induced with an unknown mechanism to promoted Bam1’s Fe resistance. Under the Cu excess condition, the CopZA efflux system would be derepressed by a bifunctional metalloregulator, CsoR, which would also derepress the uptake system, YcnJ, while YcnJ was strongly repressed by another monofunctional metalloregulator, YcnK, simultaneously in this condition, then the combined effect helped Bam1 to improve its Cu resistance. While a non-specific cation efflux, CzcD, was also activated to alleviate the cell’s Cu intoxication in this condition. When Zn was sufficient or excess, a monofunctional metalloregulator, ZurR, would repress the Zn uptake proteins, ZnuABC, then another metalloregulator, CzrA, would derepress a P-type ATPase, CadA, and a non-specific cation efflux, CzcD, for Zn export, which helped to promote the Zn resistance of Bam1. The effluxes, CadA and CzcD, also helped to improve cobalt resistance in Bam1 for Co export when Co was excess.

For the non-essential heavy metals, just some specific or non-specific exporters respond to different heavy metals were activated by the corresponding monofunctional metalloregulators. CzrA would derepress a Cd major efflux, CadA, and a non-specific cation efflux, CzcD, for Cd export when it was excess. Another regulator, ArsR, could activate the efflux pump, ArsB, which was reported for arsenite exporting mainly but could export Cd also. ArsR might sequester Cd for the improving Cd resistance of Bam1 under Cd stress. The non-specific efflux pump, PfeT, helped to alleviate Cd toxicity of Bam1 also. The important terminal oxidase in respiratory chain, Qox, also help to overcome Cd intoxication under Cd stress. When Cr was excess, the Cr ion would remove the negative effect of the regulator, ChrS, on the Cr-specific efflux pumps, ChrA and ChrB, then improved the Cr resistance of Bam1. Under As stress, the As-specific regulator, AsrR, would derepress AsrC (an arsenate reductase) and AsrB (an arsenite efflux pump), another regulator, AseR, would derepress AsrA (another arsenite efflux pump). The AsrRCB system was mainly required for As^5+^ resistance of Bam1, while both of the AsrRB and AseRA systems are required for As^3+^ resistance of Bam1. The non-specific efflux pumps, CadA and PfeT, would alleviate the lead toxicity of Bam1. The detoxification system of Hg had not been detected in Bam1.

The variation of the genes involved in heavy metal resistance and the genomic differences resulted in significant differences in heavy metal resistance between Bam1 and FZB42. The differences of the genomes between these two strains mainly included the number and arrangement of the contigs, the number of the heavy metal resistant genes within GIs and Phs, as well as the unique genes related to heavy metal resistance.

### Supplementary Information

**Additional file 1: Figure S1.** Inhibitory abilities of *B. amyloliquefaciens* Bam1 on the fungal plant pathogens. **Figure S2.** Germination and growth-promoting abilities of *B. amyloliquefaciens* Bam1 on *Euphrasia pectinate*. **Figure S3.** The MIC of Cr, Cu and Zn to *B. amyloliquefaciens* strain Bam1 and *B. velezensis* FZB42. **Figure S4.** The effect of *cadA* on the Cd resistance of *B. amyloliquefaciens* strain Bam1 and *B. subtilis* CU1065. **Table S1.** GO groups of *B. amyloliquefaciens* strain Bam1 probably involved in heavy metal resistance. **Table S2.** Genes within COG 2124 related to Cytochrome p450 in *B. amyloliquefaciens* strain Bam1. **Table S3.** Genes within DNA islands in *B. amyloliquefaciens* strain Bam1.

## Data Availability

All data generated or analyzed during this study are included in this published article.

## Footnotes

1 http://www.icama.org.cn/hysj/index.jhtml.

2 https://iaspub.epa.gov/apex/pesticides/f?p=CHEMICALSEARCH:3::::21,3,31,7,12,25:P3_XCHEMICAL_ID:1312.

3 http://www.gov.cn/foot/2014-04/17/content_2661768.htm.

4 http://www.gov.cn/foot/2014-04/17/content_2661768.htm.

5 http://soap.genomics.org.cn/.

6 http://subtiwiki.uni-goettingen.de/.

7 http://amylowiki.top/.

8 http://www.gov.cn/foot/2014-04/17/content_2661768.htm.
