# Supplementary material for: Genome sequencing of biocontrol strain Bacillus amyloliquefaciens Bam1 and further analysis of its heavy metal resistance mechanism
Source: Bioresour Bioprocess. 2022 Jul 18;9(1):74. doi: 10.1186/s40643-022-00563-x (PMC10991351; doi:10.1186/s40643-022-00563-x)
Supplement: Supplementary file 1 — Additional file 1: Figure S1. Inhibitory abilities of B. amyloliquefaciens Bam1 on the fungal plant pathogens. Figure S2. Germination and growth-promoting abilities of B. amyloliquefaciens Bam1 on Euphrasia pectinate. Figure S3. The MIC of Cr, Cu and Zn to B. amyloliquefaciens strain Bam1 and B. velezensis FZB42. Figure S4. The effect of cadA on the Cd resistance of B. amyloliquefaciens strain Bam1 and B. subtilis CU1065. Table S1. GO groups of B. amyloliquefaciens strain Bam1 probably involved in heavy metal resistance. Table S2. Genes within COG 2124 related to Cytochrome p450 in B. amyloliquefaciens strain Bam1. Table S3. Genes within DNA islands in B. amyloliquefaciens strain Bam1. [file 40643_2022_563_MOESM1_ESM.pdf]

Genome Sequencing of Biocontrol Strain *Bacillus amyloliquefaciens* Bam1 and Further Analysis of Its Heavy Metal Resistance Mechanism

Yuanchan Luo, Lei Chen, Zhibo Lu, Weijian Zhang, Wentong Liu, Yuwei Chen, Xinran Wang, Wei Du, Jinyan Luo* and Hui Wu*

* Co-corresponding authors: Hui Wu, hwu@ecust.edu.cn; Jinyan Luo, toyanzi@126.com

Additional file 1

Content

Figure S1 | Inhibitory abilities of *B. amyloliquefaciens* Bam1 on the fungal plant pathogens.

Figure S2 | Germination and growth-promoting abilities of *B. amyloliquefaciens* Bam1 on *Euphrasia pectinate*.

Figure S3 | The MIC of Cr, Cu and Zn to *B. amyloliquefaciens* strain Bam1 and *B. velezensis* FZB42.

Figure S4 | The effect of *cadA* on the Cd resistance of *B. amyloliquefaciens* strain Bam1 and *B. subtilis* CU1065.

Table S1 | The GO groups of *B. amyloliquefaciens* strain Bam1 are probably involved in heavy metal resistance.

Table S2 | The genes within COG 2124 related to Cytochrome p450 in *B. amyloliquefaciens* strain Bam1

Table S3 | The genes within DNA islands in *B. amyloliquefaciens* strain Bam1

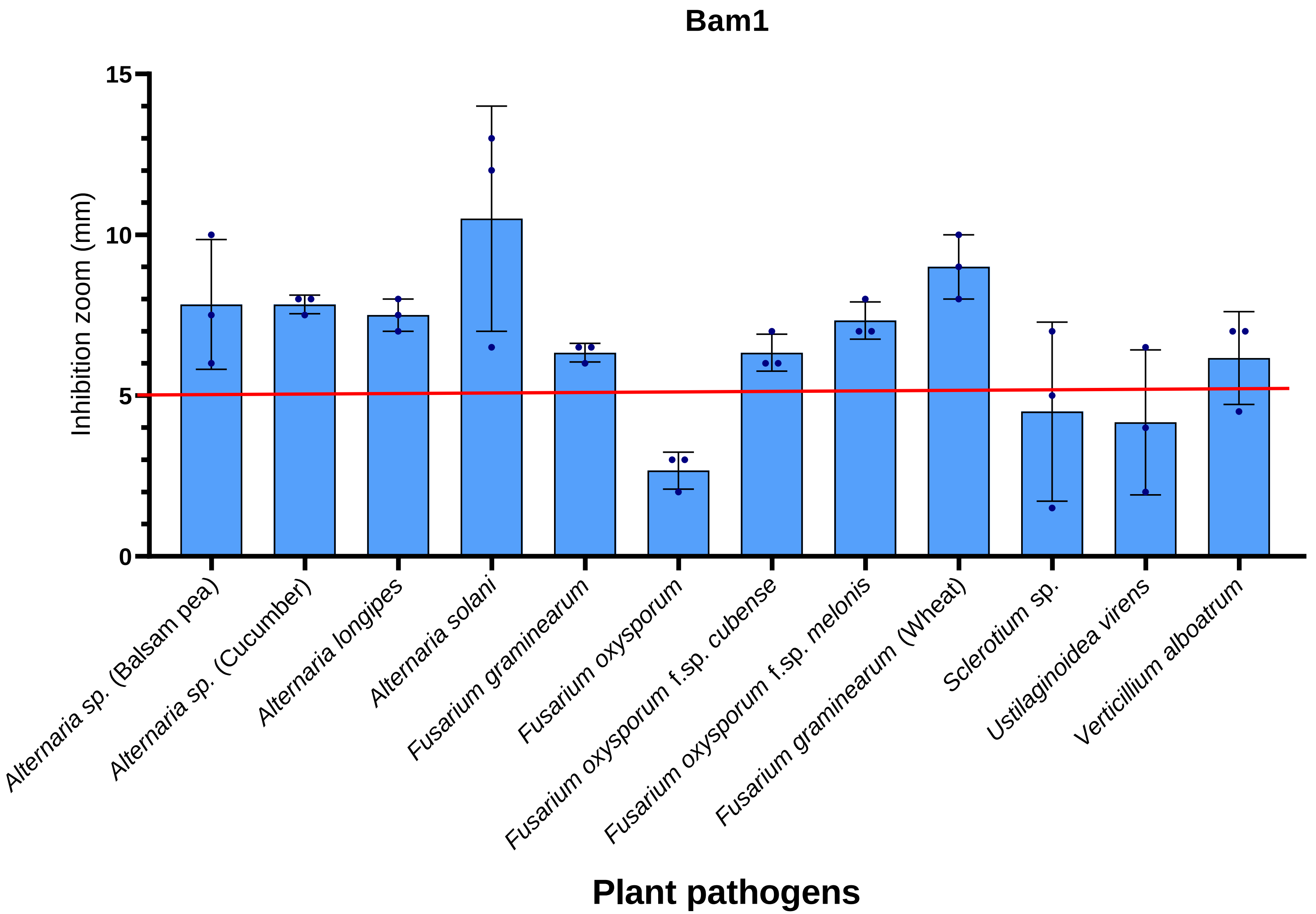

Figure S1 | Inhibitory abilities of *B. amyloliquefaciens* Bam1 on the fungal plant pathogens. All measurements are mean±SEM (n=3). The columns above the red line indicated that Bam1 possessed good inhibitory against the plant pathogens.

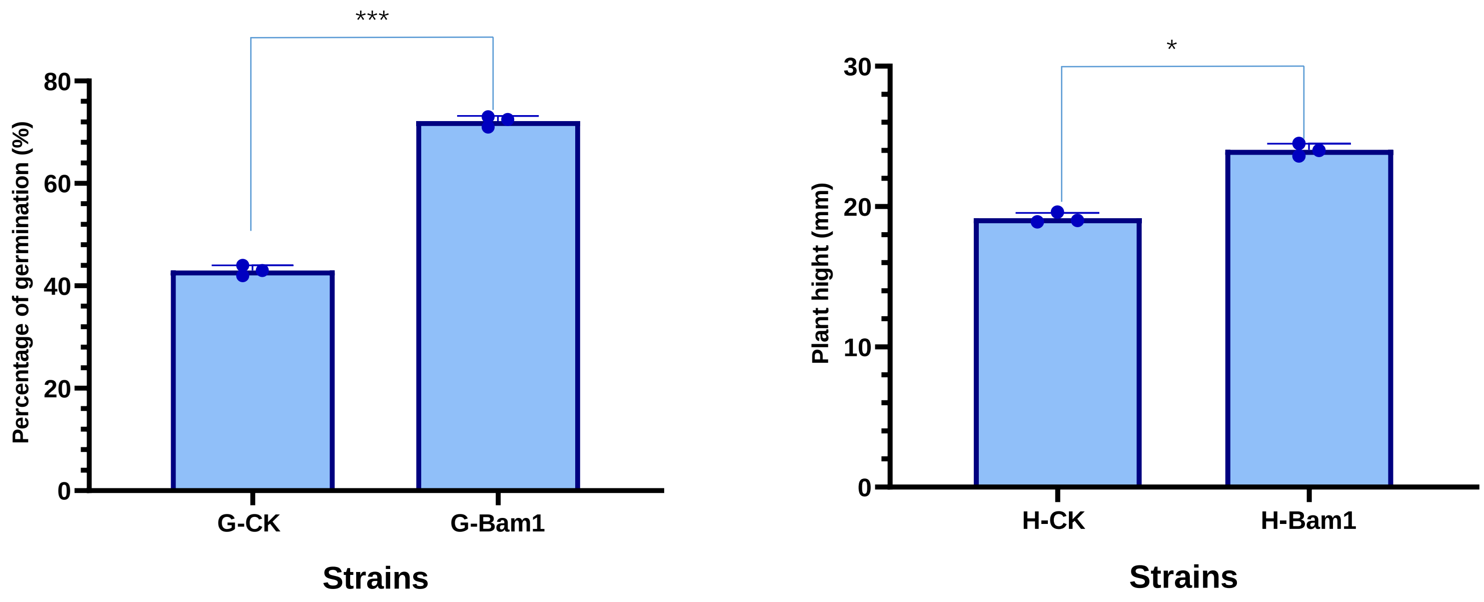

Figure S2 | Germination and growth-promoting abilities of *B. amyloliquefaciens* Bam1 on *Euphrasia pectinate*. All measurements are mean±SEM (n=3). *** represents significant difference at 0.01 level, * represents significant difference at 0.05 level**.**

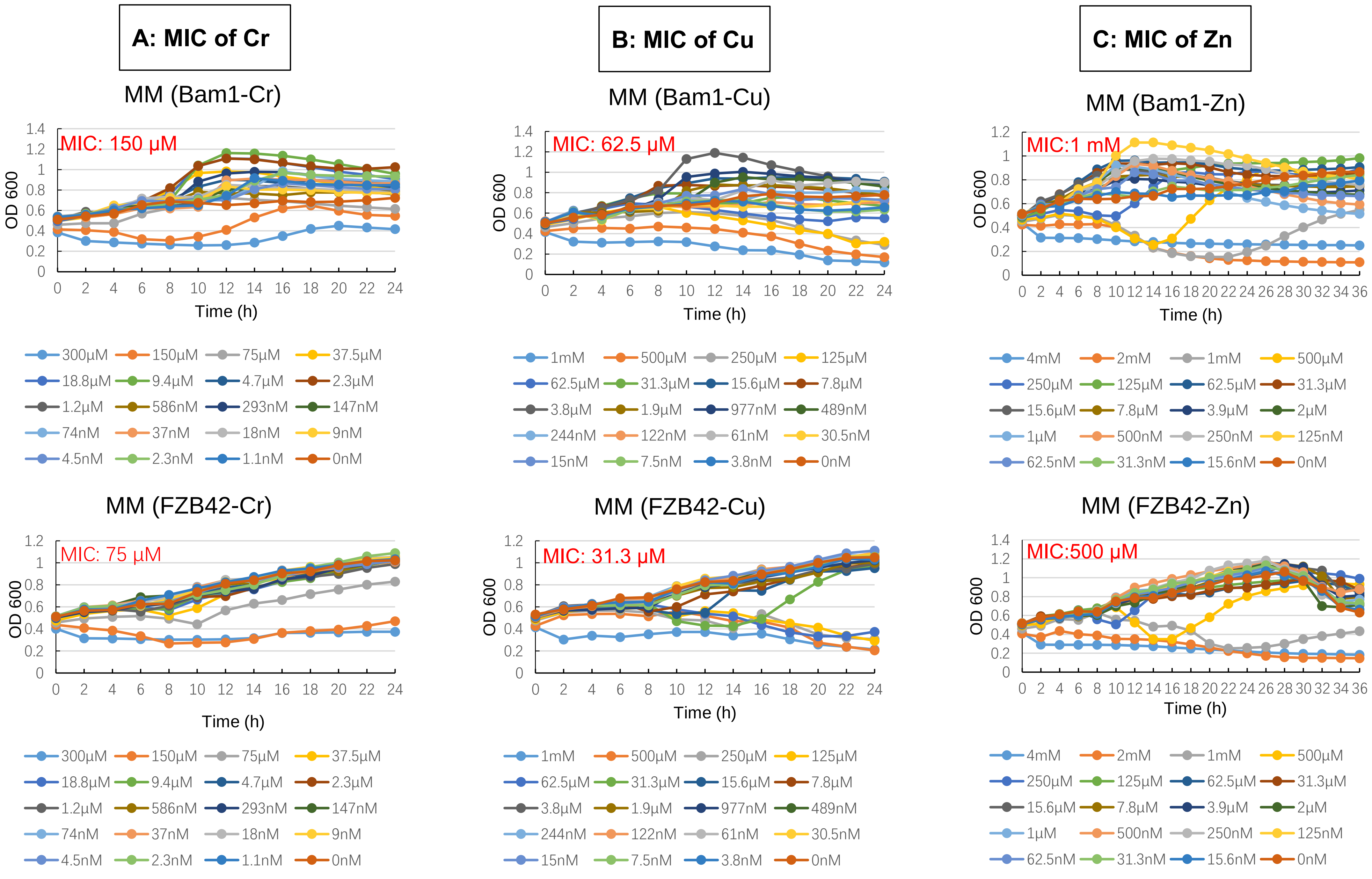

Figure S3 | The MIC of Cr, Cu and Zn to *B. amyloliquefaciens* strain Bam1 and *B. velezensis* FZB42. The concentration of Cd, Cr, Zn and Cu in the first well were 20μM, 300μM, 4mM and 1mM, respectively

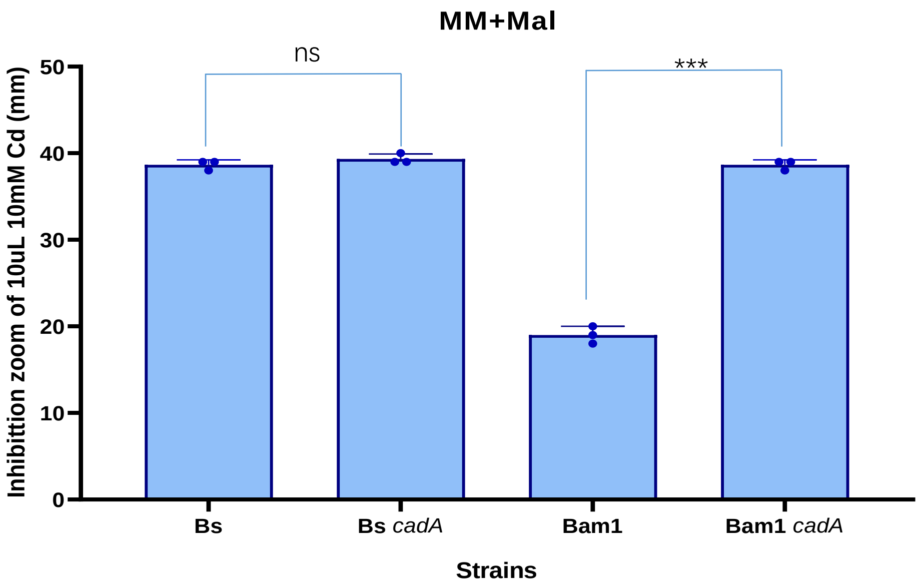

Figure S4 | The effect of *cadA* on the Cd resistance of *B. amyloliquefaciens* strain Bam1 and *B. subtilis* CU1065. All measurements are mean±SEM (n=3). *** represents significant difference (P<0.01), ns represents on statistically significant difference

Additional Tables

Table S1 | The GO groups of *B. amyloliquefaciens* strain Bam1 are probably involved in heavy metal resistance.

No.	Category	GO Description	GO ID	Gene No.	Percent(%)
1	biological_process	response to oxidative stress	GO:0006979	10	0.24
2	biological_process	response to stress	GO:0006950	10	0.24
3	biological_process	metal ion transport	GO:0030001	5	0.12
4	biological_process	ion transmembrane transport	GO:0034220	4	0.09
5	biological_process	copper ion transport	GO:0006825	2	0.05
6	biological_process	chromate transport	GO:0015703	2	0.05
7	biological_process	arsenite transport	GO:0015700	2	0.05
8	biological_process	cellular iron ion homeostasis	GO:0006879	2	0.05
9	biological_process	cellular manganese ion homeostasis	GO:0030026	1	0.02
10	biological_process	respiratory electron transport chain	GO:0022904	1	0.02
11	biological_process	response to copper ion	GO:0046688	1	0.02
12	biological_process	response to arsenic-containing substance	GO:0046685	1	0.02
13	biological_process	ferrous iron transport	GO:0015684	1	0.02
14	cellular_component	ATP-binding cassette (ABC) transporter complex	GO:0043190	12	0.28
15	cellular_component	respiratory chain	GO:0070469	4	0.09
16	cellular_component	high-affinity iron permease complex	GO:0033573	1	0.02
17	molecular_function	metal ion binding	GO:0046872	124	2.94
18	molecular_function	zinc ion binding	GO:0008270	67	1.59
19	molecular_function	transporter activity	GO:0005215	61	1.45
20	molecular_function	ATPase activity	GO:0016887	53	1.26
21	molecular_function	iron ion binding	GO:0005506	26	0.62
22	molecular_function	manganese ion binding	GO:0030145	15	0.36
23	molecular_function	iron-sulfur cluster binding	GO:0051536	15	0.36
24	molecular_function	ATPase activity, coupled to transmembrane movement of substances	GO:0042626	12	0.28

25	molecular_function	cation transmembrane transporter activity	GO:0008324	12	0.28
26	molecular_function	copper ion binding	GO:0005507	8	0.19
27	molecular_function	nickel cation binding	GO:0016151	4	0.09
28	molecular_function	antioxidant activity	GO:0016209	4	0.09
29	molecular_function	cation-transporting ATPase activity	GO:0019829	3	0.07
30	molecular_function	NAD(P)H dehydrogenase (quinone) activity	GO:0003955	2	0.05
31	molecular_function	chromate transmembrane transporter activity	GO:0015109	2	0.05
32	molecular_function	ferredoxin-NADP+ reductase activity	GO:0004324	2	0.05
33	molecular_function	cobalt ion binding	GO:0050897	2	0.05
34	molecular_function	metal ion transmembrane transporter activity	GO:0046873	1	0.02
35	molecular_function	cation binding	GO:0043169	1	0.02
36	molecular_function	ion channel activity	GO:0005216	1	0.02
37	molecular_function	ion transmembrane transporter activity	GO:0015075	1	0.02
38	molecular_function	iron ion transmembrane transporter activity	GO:0005381	1	0.02

Table S2 | The genes within COG 2124 related to Cytochrome p450 in *B. amyloliquefaciens* strain Bam1

Gene ID	Gene Name	COG ID	COG Description	Gene Description	COG Type Description	COG Category
gene0738	*cypD_E*	COG2124	Cytochrome p450	NADPH--cytochrome P450 reductase	Secondary metabolites biosynthesis, transport and catabolism	METABOLISM
gene1149	--	COG2124	Cytochrome p450	Cytochrome p450	Secondary metabolites biosynthesis, transport and catabolism	METABOLISM
gene1356	--	COG2124	Cytochrome p450	Putative monooxygenase	Secondary metabolites biosynthesis, transport and catabolism	METABOLISM
gene1872	*pksS*	COG2124	Cytochrome p450	Cytochrome p450	Secondary metabolites biosynthesis, transport and catabolism	METABOLISM
gene1908	--	COG2124	Cytochrome p450	Cytochrome p450	Secondary metabolites biosynthesis, transport and catabolism	METABOLISM
gene2003	*bioI*	COG2124	Cytochrome p450	Cytochrome p450	Secondary metabolites biosynthesis, transport and catabolism	METABOLISM

Table S3 | The genes within DNA island in *B. amyloliquefaciens* strain Bam1 (except for hypothetical proteins).

DNA island	Gene	Product or function
GI01/Ph01	gene1243	phage portal protein
Ph01	gene1278	terminase
Ph01	gene1279	phage portal protein
GI02	gene1303	phage related protein
GI02/Ph01	gene1304	phage holin
Ph01	gene1324	putative bacteriophage protein
GI02/Ph01	gene1327	phage coat protein
GI04/Ph04	gene3226	autolysin
GI05	gene3370	holin
GI05	gene3389	type IV secretion protein Rhs
GI05	gene3390	portal protein
GI07	gene4300	MarR family transcriptional regulator
GI07	gene4303	GntR family transcriptional regulator
